# Key factors and serial mediation analysis of anxiety and depression among university students: a cross-sectional study

**DOI:** 10.3389/fpsyt.2026.1809332

**Published:** 2026-05-19

**Authors:** Tan Jiang, Shijia Zhang, Deyou Jiang

**Affiliations:** 1 Faculty of Arts and Social Sciences, Hong Kong Baptist University, Hong Kong, China; 2 First Affiliated Hospital, Heilongjiang University of Chinese Medicine, Harbin, Heilongjiang, China; 3 Heilongjiang University of Chinese Medicine, Harbin, Heilongjiang, China

**Keywords:** adverse childhood experiences, anxiety, depression, family interaction, sleep, social support, university students

## Abstract

**Background:**

Anxiety and depressive symptoms are prevalent among university students; however, their risk factors are unlikely to operate in isolation. Instead, they may be organized in a multilevel structure that progressively approaches emotional outcomes, spanning from distal adversity exposure and the current family relational context, through resource pathways, to proximal functional status. This study aimed to identify the key risk factors for anxiety/depressive states among university students and to examine whether a sequential statistical association structure exists among childhood adversity exposure, family interaction, social support, and sleep status.

**Methods:**

A cross-sectional online survey was conducted in seven universities in Harbin (Sep–Nov 2025; N = 9, 796). Depressive and anxiety states were assessed by PHQ-9 (≥5) and GAD-7 (≥5). Family cohesion and adaptability (FACES II-CV), the Parent–Adolescent Communication Scale (PACS) and the Social Support Rating Scale (SSRS) were administered, and childhood harmful exposure, family conflict, recent negative life events, lifestyle, and sleep status were measured. Hierarchical logistic regression and LightGBM, MLP, SVM models were used for analysis, with SHAP applied to identify key correlates, and serial mediation models were used to examine the sequential statistical associations among variables.

**Results:**

The prevalence of depressive and anxiety states was 19.7% and 19.5%. For both outcomes, sleep status showed the strongest and most stable association (depression OR = 1.431; anxiety OR = 1.335). childhood harmful exposure, problem communication, family conflict, and recent major negative life events were consistent risk correlates for both outcomes, whereas social support was a stable protective factor. Machine learning results showed that LightGBM achieved the best overall performance. The overall risk profiles were similar across the two outcomes; however, childhood harmful exposure contributed more to depressive states, whereas problematic communication and family conflict were more prominent in anxiety states. Serial mediation analysis further showed that a sequential statistical association structure, composed of negative family interactions, social support, and sleep status, existed between childhood harmful exposure and anxiety/depressive states, with sleep problems showing the largest indirect effect.

**Conclusion:**

Anxiety and depressive states in university students exhibited a sequential statistical association structure that progressively approached emotional outcomes, extending from early adverse experiences and current negative family interactions, through insufficient social support, to proximal sleep impairment. Sleep problems were a shared key proximal correlate of both outcomes. In addition, although anxiety and depression shared highly similar risk profiles, anxiety was more closely related to current relational tension and real-life stress, whereas depression was more closely related to long-term cumulative adverse experiences. Prioritizing assessment and stratified intervention around the three key domains of sleep, negative family interactions, and social support may help improve the specificity and efficiency of mental health services in universities.

## 1 Introduction

Anxiety and depression are among the most common mental health problems worldwide and impose one of the heaviest burdens of disease. The World Health Organization has noted that the core feature of mental disorders is a clinically significant disturbance in an individual’s cognition, emotion regulation, or behavior, accompanied by marked distress and functional impairment, among which anxiety and depressive disorders are the most common types. Anxiety disorders are mainly characterized by persistent excessive worry, heightened tension and vigilance, and related somatic arousal responses; depressive disorders are mainly characterized by persistent depressed mood and diminished interest or pleasure, and are often accompanied by abnormalities in sleep, appetite, energy, and cognitive function (1). The latest Global Burden of Disease report indicates that approximately 359 million people worldwide are living with anxiety disorders and 280 million with depressive disorders (2). By comparison, anxiety and depression are more prominent among university students, who are in a critical transitional stage from late adolescence to early adulthood and are exposed to multiple stressors, including academic pressure, interpersonal adjustment, employment expectations, and lifestyle changes (3). Data from the WMH-ICS cross-national survey show that the prevalence of depression- and anxiety-related disorders among university students is approximately 18.5% and 16.7%, respectively (4). Emotional problems during this stage of youth often have cumulative effects (5). A large body of research has confirmed that anxiety/depression is closely associated with adverse outcomes such as impaired academic functioning, difficulties in interpersonal adaptation, and increased risks of self-harm and suicide (6–10). Therefore, a more refined identification and interpretation of their risk structure is of important public health and intervention value.

Existing research suggests that the occurrence of depression and anxiety is not attributable to a single factor, but rather reflects a stress process characterized by cumulative development over time and stepwise transmission, in which multiple stressors gradually accumulate and jointly affect emotional health outcomes through individuals’ coping patterns, psychosocial resources, and proximal psychosomatic functional status (11–13). This is consistent with the core perspective of the Stress Process Model (SPM) (14, 15). SPM, proposed by Pearlin et al. (14), was developed to explain how stress affects health outcomes through a series of interrelated psychosocial mechanisms. The model generally comprises three core components: sources of stress, such as life events and chronic stressors; mediating or resource systems, such as self-concept, coping styles, and social support; and stress outcomes, namely mental or physical health problems such as anxiety and depression. The SPM emphasizes that stress does not occur in isolation; rather, within a broader social-structural context, it gradually influences health outcomes through processes involving resource depletion, coping regulation, and functional impairment. At the same time, inequalities in stress exposure and access to resources across individuals in different social circumstances may also lead to differences in mental health risk (16). Previous studies have provided segmented evidence regarding stress exposure, psychosocial resources, proximal functional status, and emotional outcomes across different populations and contexts (17–20). Guided by the stepwise transmission pathway emphasized by SPM—namely, sources of stress, resource/mediating systems, and health outcomes—and informed by existing evidence on emotional problems in university students, the present study identified four representative core domains: distal adversity exposure, family relational context, resource pathways, and proximal functional status, in order to examine the multilevel stress-process structure underlying depressive risk and anxiety states in university students.To render this framework empirically testable, these four domains were operationalized into four core variables: childhood harmful exposure, family interaction, social support, and sleep status. These variables collectively cover sources of early vulnerability, current relational context, resource reserves, and the proximal functional manifestations most directly related to emotional outcomes, thereby constituting the core variable system for the subsequent hypothesis testing in this study.

### 1.1 Childhood harmful exposure

Childhood harmful exposure generally refers to persistent harmful treatment or deficiencies in care experienced by an individual before the age of 18 within the primary caregiving environment. It includes emotional, physical, and sexual abuse, as well as long-term caregiving deprivation and threatening family interactions. Existing research has relatively consistently shown that childhood harmful exposure is significantly associated with subsequent risks of internalizing problems such as depression and anxiety, and that these associations often exhibit cumulative effects (21, 22). Among these experiences, physical abuse, sexual abuse, emotional abuse, and neglect/caregiving deprivation appear to be more closely linked to outcomes such as depression and anxiety (23). Therefore, from a stress-process perspective, childhood harmful exposure is not only an important manifestation of early adversity, but also a key distal source of subsequent emotional vulnerability.

Regarding the underlying mechanisms, existing studies have mainly focused on three aspects. First, early harmful exposure may significantly increase individuals’ sensitivity to subsequent stress, making them more prone to hypervigilance, negative expectations, and anxiety/depressive risk under stress (24). Second, harmful exposure may increase the risk of internalizing problems by altering emotional processing and self-related cognition (25, 26). Third, early adversity may lead to dysregulation of the hypothalamic–pituitary–adrenal (HPA) axis and disruption of cortisol rhythms, thereby making individuals more likely to exhibit excessive or delayed stress responses under subsequent stress (27), these long-term physiological and psychological alterations may not only be reflected in elevated risks of anxiety and depression, but may also become embedded in subsequent relational adaptation and everyday functional status. Specifically, heightened stress sensitivity, strengthened negative self-cognition, threat-related vigilance, and dysregulated stress responses may make individuals more likely to develop distrustful, hypervigilant, avoidant, or defensive interaction patterns in subsequent interpersonal contexts, while also weakening their ability to perceive and utilize supportive relationships (28, 29). In addition, sustained hyperarousal and neuroendocrine dysregulation may further impair sleep maintenance and restorative functioning (30). Taken together, this suggests that childhood harmful exposure is not an isolated distal risk factor, but rather one that may continue to shape emotional health outcomes during the university years through multiple subsequent domains, including family relationships, social support, and sleep status. Among these, its link with family interaction patterns appears to be the most direct. On the one hand, childhood harmful exposure largely occurs within the primary caregiving system and therefore has an inherent contextual continuity with emotional responses, communication styles, and conflict patterns within the family environment. On the other hand, attachment insecurity, difficulties in emotion regulation, and negative relational expectations shaped by early adversity are also most likely to be maintained, reinforced, or modified first within the current family relational context. Therefore, to further understand how childhood harmful exposure remains associated with depressive risk and anxiety states among university students, it is necessary to examine its continued manifestation in current family interaction.

### 1.2 Family interaction

Based on the foregoing evidence, family interaction should not be regarded as an independent background factor detached from childhood harmful exposure; rather, it represents an important relational context through which the effects of distal adversity are carried forward into the current environment. The quality of family interaction may buffer, amplify, or consolidate individual vulnerability. Accordingly, within the SPM framework, family interaction not only constitutes a key relational context for current emotional risk, but also serves as a critical intermediate domain for understanding how distal adversity is transmitted to subsequent resource acquisition and emotional health outcomes.

In the literature on family systems and family environment, family interaction is generally organized around three interrelated core process dimensions. The first is family functioning, referring to the cohesion/intimacy and adaptability/flexibility of the family system (31). The second is family communication, referring to the ways in which family members express emotions, discuss problems, and respond to one another. The third is family conflict, referring to highly arousing relational tension such as blame, quarrels, yelling, and even more intense confrontation (32). Previous studies have repeatedly confirmed that family interaction is stably associated with depressive and anxiety-related symptoms (33–35). Further research has shown that family functioning may be associated with depression through coping styles (36), whereas family functioning and social support may be associated with anxiety through process variables such as sense of coherence and self-esteem (37). In addition, studies have found that individuals in negative family environments are more likely to develop rumination, threat interpretation bias, and ineffective coping styles (36, 37). Taken together, these findings suggest that family functioning may jointly influence internalizing symptoms through relationship quality, coping styles, and psychological resources.

More specifically, family dysfunction, impaired communication, and frequent conflict may make individuals more likely to develop rumination, threat interpretation bias, and ineffective coping styles, while also weakening process-related resources closely linked to psychological regulation, such as sense of coherence and self-esteem (32, 37, 38). At the same time, such long-term relational imbalance may further affect individuals’ perceptions of support availability, their trust in relational resources, and their ability to identify, integrate, and utilize supportive resources (39). This suggests that family interaction may continue to shape emotional health outcomes during the university years through multiple subsequent domains, including social support and proximal functional status. Among these, its connection with social support appears to be the most direct. On the one hand, family interaction itself constitutes one of the most central and enduring sources of relational experience, and its quality directly influences individuals’ basic perceptions of support availability (40). On the other hand, coping orientations, self-esteem, and sense of coherence shaped by family interaction are also most likely to be reflected first in how individuals accept, seek, and utilize supportive resources (37). Therefore, it is necessary to further examine the role of social support in relation to anxiety/depressive states, as well as its continued manifestation within the stress process.

### 1.3 Social support

Social support refers to the emotional, instrumental, informational, and belonging resources that individuals can obtain and subjectively perceive under stressful circumstances. It determines whether individuals possess mobilizable external resources to share burdens, revise threat appraisals, and maintain emotional regulation when facing stress. Classical research has shown that social support may exert both a direct protective effect on mental health and a “stress-buffering” effect that attenuates the transformation of stress into pathological symptoms (41, 42). More recent studies have further suggested that, in the development of anxiety, social support may also operate by enhancing communicative adaptability and reducing perceived social threat (43). Taken together, these findings indicate that social support is not merely a general background variable, but rather a key protective resource within the stress process; accordingly, it is often positioned at the core of the “resource pathway” in psychological research. When support is insufficient, or when it cannot be stably perceived and effectively utilized, stress is more likely to be transmitted to the level of everyday functioning in the form of persistent tension, repetitive rumination, and impaired recovery (44). As an important proximal functional domain for restorative capacity, arousal regulation, and the maintenance of emotional homeostasis, sleep is particularly susceptible to the joint influence of stress and resources. Consequently, whether the resource pathway functions effectively is often first manifested in sleep maintenance, sleep quality, and restorative functioning (45).

### 1.4 Sleep status

Sleep status is a multidimensional construct involving the overall manifestation of abnormalities in sleep initiation, sleep maintenance, sleep duration, and daytime restorative functioning, and represents one of the most typical proximal functional states. During the university years, individuals are confronted with multiple changes, including greater autonomy over daily schedules, increased academic demands, greater exposure to electronic media, and instability in social rhythms, making sleep problems one of the most common functional manifestations among young people and one of the manifestations most likely to be intertwined with emotional problems (46). Existing studies have found that, among university students, sleep problems often co-occur significantly with anxiety and depression: the poorer the sleep quality, the greater the burden of psychological distress and internalizing symptoms, and the higher the subsequent risk of depression and anxiety (47, 48). Further studies have shown that sleep impairment may promote the onset and maintenance of internalizing symptoms such as anxiety and depression by weakening emotion regulation (49, 50), disrupting the processing of threat cues and the updating of safety information (51, 52), and reducing daily restoration and socioemotional functioning (53). These findings further support the key role of sleep in the development and maintenance of internalizing symptoms. In addition, the heightened stress sensitivity and dysregulated stress responses associated with childhood harmful exposure, the rumination, threat interpretation bias, and ineffective coping reinforced by negative family interaction, and the insufficient stress buffering and constrained resource utilization associated with low social support may all, through mechanisms such as sustained cognitive arousal, circadian disruption, and impaired recovery, be further manifested as difficulties in sleep maintenance, reduced sleep quality, and impaired daytime restorative functioning. Accordingly, sleep not only represents a concentrated manifestation of the stress process at the level of proximal functioning, but may also serve as an important carrier linking the aforementioned multilevel risk factors to anxiety/depressive outcomes; for this reason, it was included in the present study as a key variable.

In addition, recent major negative life events, as acute real-life stressors, and lifestyle factors, as modifiable behavioral background variables, have also been repeatedly shown to be associated with anxiety/depression among university students. The former may reflect the real-life stress burden recently experienced by individuals (54), whereas the latter may further affect emotional health through circadian rhythms, self-regulation, and physical status (55). Therefore, both are often included in analyses as background variables of real-life stress.

Although previous studies have separately confirmed stable associations of childhood harmful exposure, family interaction, social support, and sleep status with anxiety/depression, several limitations remain. Most studies have focused on a single risk factor or a single mediating mechanism, with relatively few systematically integrating distal adversity exposure, family relational context, resource pathways, and proximal functional status within a unified theoretical framework; consequently, it remains difficult to comprehensively elucidate the multilevel psychosocial structure underlying anxiety/depression risk among university students (56–58). In addition, the existing literature remains insufficient in its discussion of the commonalities and differences in the risk structures of anxiety and depression, particularly with regard to parallel comparisons within the same variable system (56, 59, 60). Furthermore, the identification of key risk factors and the examination of sequential association structures among variables have largely been conducted separately, making it difficult to simultaneously address the relative importance of risk factors and their structural associations within a unified framework (56, 61, 62).

Based on the above analysis, the present study focused on university students and, within the SPM framework, constructed a research variable system around four core domains: distal adversity exposure, family relational context, resource pathways, and proximal functional status. These domains were operationalized as childhood harmful exposure, family interaction, social support, and sleep status, respectively, while recent major negative life events and lifestyle factors were simultaneously included as background variables of real-life stress. On the one hand, the study examined the strength of association and relative importance of variables at different levels with depressive risk and anxiety states in order to identify the key correlates of emotional risk among university students. On the other hand, following the theoretical logic of SPM, it further tested whether a sequential statistical association structure, consistent with the theoretical ordering proposed by SPM, existed among childhood harmful exposure, family interaction, social support, and sleep status. In addition, depressive risk and anxiety states were analyzed separately as outcomes to compare their common risk factors and key points of divergence.

Based on SPM and the above literature review, the present study proposed the following hypotheses.

H1: Among the multilevel correlates, sleep status is expected to show strong associations with both depressive and anxiety states among university students, and to demonstrate relatively high importance in the ranking of variable contributions.

H2: Although depressive and anxiety states share several core correlates, differences are expected in the strength of associations and the ranking of variable contributions between the two outcomes.

H3: A sequential indirect association consistent with the logic of SPM is expected between childhood harmful exposure and depression risk/anxiety state among university students. Specifically, more negative family interaction, lower social support, and more severe sleep problems may jointly constitute the statistical association structure linking distal adversity and emotional outcomes.

## 2 Methods

### 2.1 Study design and participants

This study employed a cross-sectional questionnaire survey design. The survey was conducted from September to November 2025 in seven universities located in Harbin, Heilongjiang Province, China: Heilongjiang University of Chinese Medicine, Heilongjiang University, Harbin University of Science and Technology, Harbin Institute of Technology, Harbin Normal University, Harbin Finance University, and Harbin Sport University. The study population consisted of undergraduate students enrolled at these universities. Considering the need for multi-university sample coverage, standardized questionnaire administration procedures, and unified data collection protocols, the survey was conducted centrally through an online platform.

The questionnaire was distributed and collected through the Wenjuanxing platform. Recruitment was facilitated with the assistance of university counselors and teachers, who forwarded the survey link to the corresponding class group chats. After reading the study description and providing electronic informed consent online, students independently decided whether to participate and completed the questionnaire voluntarily and without coercion. Therefore, the sample in this study represents a voluntary non-probability sample recruited through university-assisted channels. A total of 10, 841 questionnaires were collected, of which 9, 796 valid questionnaires were included in the analysis after quality control, yielding a valid questionnaire rate of 90.36%.

Before the formal survey, the research team conducted a small-scale pilot test to assess the feasibility of the questionnaire and revised it accordingly, resulting in the final version. The formal questionnaire contained 108 items, with an average completion time of approximately 15 minutes. To ensure data completeness and comparability, the online questionnaire was configured with a “mandatory response” setting, meaning that respondents could not proceed to the next item until the current item had been completed. To reduce the risk of duplicate responses, the survey platform was also set to allow only one submission per account.

**Inclusion criteria:**

1. Undergraduate students enrolled at the seven universities listed above;
2. Voluntary provision of electronic informed consent after reading the study description;
3. Ability to independently understand the questionnaire content and complete the online survey.

**Exclusion criteria:**

1. Records in which the questionnaire was not finally submitted;
2. Records in which the self-reported university, educational level, or enrollment status did not meet the study inclusion criteria;
3. Records with an age outside the range of 17–24 years;
4. Records showing obvious internal logical contradictions or inconsistencies across responses;
5. Records that failed the two attention-check items embedded in the questionnaire;
6. Records with abnormal response durations, defined as completion times shorter than 420 seconds or longer than 2400 seconds.

**Figure 1:**
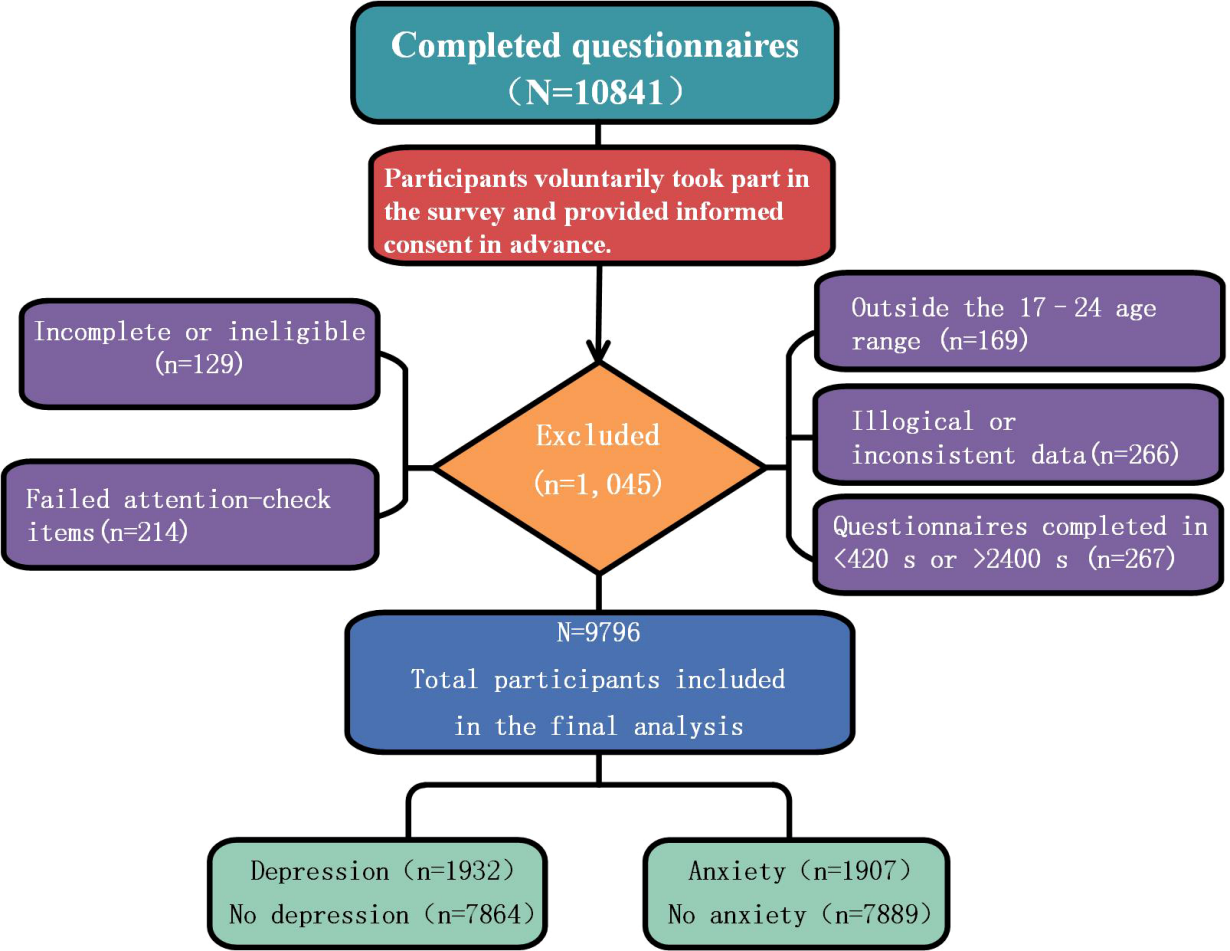
Participant flow diagram.

Data cleaning was implemented stepwise according to a pre-specified quality control procedure. First, eligibility screening was conducted for all returned records. Questionnaires that were not finally submitted, or in which the self-reported university, educational level, or enrollment status did not meet the study inclusion criteria, were excluded (n = 129). Second, a plausibility check was performed for the age variable. As all questionnaire items were answered using predefined options except for age, which was manually entered, age was the only variable subjected to numerical outlier screening in this study. Considering that the age of students in Chinese higher education is typically concentrated between 18 and 22 years and that the standard duration of undergraduate education is generally 4 to 5 years, the range of 17–24 years was defined as the reasonable age range for full-time undergraduate students (63). Records outside this range were excluded (n = 169). Third, based on pre-established logical consistency rules, records showing obvious internal logical contradictions or inconsistencies across responses were identified and excluded (n = 266). During this stage, the research team also conducted an auxiliary quality check of response patterns in continuous scale item sets, examining whether multiple conceptually similar items showed extremely low variability in responses. This indicator was used as a reference signal for identifying potential low-quality responses; however, it was not used as an independent exclusion criterion. Final exclusions were determined based on the predefined criteria of logical inconsistency, attention-check failure, and abnormal response duration.Subsequently, according to the results of the two attention-check items embedded in the questionnaire, records that failed these checks were excluded (n = 214). Finally, based on the distribution of response times, questionnaires completed in less than 420 seconds or more than 2400 seconds were classified as having abnormal completion times and were excluded (n = 267). After the above quality control procedures, a total of 1, 045 questionnaires were excluded, resulting in 9, 796 valid samples included in the final statistical analysis. The participant inclusion and exclusion process is shown in **Figure 1**.

For incomplete responses, questionnaires that were not finally submitted were regarded as invalid records and were removed during the data cleaning stage. Because the formal survey used a mandatory-response setting for all items, questionnaires that were formally submitted contained no item-level missing values. Therefore, the final analysis adopted a complete-case analysis approach, and no missing data imputation was performed. Regarding outliers and abnormal records, entries with ages outside the predefined reasonable range were treated as numerical outliers. Records showing obvious internal logical contradictions, failure of the attention-check items, or abnormal response durations were regarded as low-quality questionnaires and were excluded during data cleaning. Extremely low variability in responses within continuous scale item sets was used only as an auxiliary signal for quality checking and was not used as an independent exclusion criterion.

### 2.2 Measures

#### 2.2.1 Outcomes

The outcome variables were depressive status and anxiety status, measured using the PHQ-9 (Patient Health Questionnaire-9) and GAD-7 (Generalized Anxiety Disorder-7). The PHQ-9 was developed by Kroenke et al. as a brief self-report instrument based on the DSM-IV diagnostic criteria for depressive disorders and is used to assess the frequency and severity of depressive symptoms over the past two weeks (64). The scale consists of nine items corresponding to core depressive symptoms, including diminished interest, depressed mood, sleep problems, fatigue, changes in appetite, low self-evaluation, difficulty concentrating, psychomotor changes, and thoughts of death or self-harm. Each item is scored on a four-point scale: 0 indicates “not at all, “ 1 indicates “several days, “ 2 indicates “more than half the days, “ and 3 indicates “nearly every day.” The total score ranges from 0 to 27, with higher scores indicating more severe depressive symptoms (64). In this study, the PHQ-9 was used to characterize the level of depressive symptoms and the risk status among university students. The scale demonstrated good internal consistency in the present sample (Cronbach’s α = 0.912). The GAD-7 was developed by Spitzer et al. (65). It was originally designed for screening generalized anxiety disorder and is now widely used to assess the frequency and severity of anxiety symptoms over the past two weeks. The scale consists of seven items covering key anxiety-related symptoms, including feeling nervous or anxious, inability to control worrying, excessive worrying, difficulty relaxing, restlessness, irritability, and fear that something awful might happen. Each item is scored on a four-point scale: 0 indicates “not at all, “ 1 indicates “several days, “ 2 indicates “more than half the days, “ and 3 indicates “nearly every day.” The total score ranges from 0 to 21, with higher scores indicating more severe anxiety symptoms (65). In this study, the GAD-7 was used to characterize the level of anxiety symptoms and the risk status among university students. The scale demonstrated good internal consistency in the present sample (Cronbach’s α = 0.918).

In this study, PHQ-9 ≥ 5 and GAD-7 ≥ 5 were used as the cutoff thresholds for depressive risk and anxiety risk, respectively. The rationale for adopting these thresholds is twofold. First, in the original validation studies of the PHQ-9 and GAD-7, a score of 5 was defined as the starting point for mild symptom severity (64, 65). Second, subsequent studies have shown that, across different populations, a cutoff of 5 demonstrates good sensitivity, specificity, and overall screening performance (66–68). Therefore, the use of ≥5 in this study was intended to more sensitively identify mild or higher levels of emotional symptom burden, early risk states, and subthreshold psychological problems among university students, rather than to define clinically diagnosed cases of depressive or anxiety disorders.

#### 2.2.2 Predictors

This study adopted SPM as the theoretical framework and constructed a system of explanatory variables around four core domains: distal adversity exposure, family relational context, resource pathways, and proximal functional status. Demographic and family background variables, health background, recent major negative life events, and lifestyle factors were simultaneously included as background and control variables. Specifically, the explanatory variables included demographic and family background, health background, childhood harmful exposure, family interaction, social support, recent major negative life events, lifestyle-related behaviors, and sleep status.

Demographic and family background variables included sex, age (in years), only-child status, co-residence with parents, family economic status, parental marital status, and the highest educational level and occupational type of both father and mother. The coding of these categorical variables is presented in **Table 1**. These variables were primarily used to characterize the basic sociodemographic characteristics and family background differences of the sample and were included as baseline covariates in subsequent analyses to control for the potential influence of developmental background and family social structure on depression risk and anxiety state.Health background variables included the presence of chronic physical diseases and a family history of mental disorders among first-degree relatives. These variables were included to control for the potential influence of prior health conditions and familial vulnerability to mental disorders on depression risk and anxiety state (69, 70).

**Table 1:** Definitions and coding of study variables.

Variables	Assignment
Sex	1 = Male; 2 = Female
Only-child status	1 = No; 2 = Yes
Living arrangement with parents	1 = Living together; 2 = Not living together but meeting weekly; 3 = Not living together and meeting less than once per month
Parental marital status	1 = Married; 2 = Divorced/Separated; 3 = Widowed
Father’s highest educational level	1 = Junior high school or below; 2 = Senior high school/technical secondary school; 3 = Bachelor’s degree; 4 = Master’s degree or above
Mother’s highest educational level	1 = Junior high school or below; 2 = Senior high school/technical secondary school; 3 = Bachelor’s degree; 4 = Master’s degree or above
Father’s occupational category	1 = Enterprise employee; 2 = Civil servant/Public institution staff; 3 = Self-employed; 4 = Agriculture/Migrant labor; 5 = Other
Mother’s occupational category	1 = Enterprise employee; 2 = Civil servant/Public institution staff; 3 = Self-employed; 4 = Agriculture/Migrant labor; 5 = Other
Family economic status	1 = Financial difficulty; 2 = Average; 3 = Affluent
History of chronic physical disease	1 = No; 2 = Yes
Family history of mental disorders (first-degree relatives)	1 = No; 2 = Yes
Average daily screen time	1=<2h;2 = 2-4h;3 = 4-6h;4 = 6-8h;5 = 8-10h;6 = 10-12h;7=>12h
Regular physical activity	1 = ≥ 3 times/week; 2 = 1–2 times/week; 3 = Rarely
Caffeine intake	1 = ≥ 1 cup/day; 2 = Occasional; 3 = None
Alcohol consumption	1 = Never; 2 = Occasional; 3 = Daily
Smoking status	1 = Never; 2 = Occasional; 3 = Daily
Recent major negative life events	1 = No; 2 = Yes
Anxiety status	1 = No; 2 = Yes
Depressive status	1 = No; 2 = Yes

Childhood harmful exposure served as the indicator of distal adversity exposure in this study. This indicator was developed with reference to the core trauma dimensions covered by the Childhood Trauma Questionnaire–Short Form (CTQ-SF). The standard CTQ-SF, developed by Bernstein et al. based on the Childhood Trauma Questionnaire, contains 28 items, among which 25 clinical items primarily assess five dimensions: emotional abuse, physical abuse, sexual abuse, emotional neglect, and physical neglect (71).In the present study, childhood harmful exposure was assessed using the following seven items: whether, before the age of 18, the respondent had experienced physical punishment by parents (e.g., being slapped or beaten with objects); whether the respondent had often experienced hunger, inadequate clothing, or delayed medical care due to parental neglect; whether parents frequently used demeaning statements such as “you are not as good as others” or “it would be better without you”; whether the respondent had ever felt extreme fear or helplessness because of parental behavior; whether parents had ever threatened to abandon or send the respondent away; whether the respondent had experienced sexual victimization (including non-contact sexual harassment); and whether the respondent had often been publicly humiliated by parents. These items primarily correspond to indicators of childhood trauma such as physical abuse, physical neglect, emotional abuse, and sexual abuse (72), and more directly reflect explicit harmful exposure, relational harm, and substantial caregiving deprivation during childhood.Compared with the full measurement of the five trauma dimensions in the CTQ-SF, the present study prioritized items with stronger behavioral specificity that could be directly reported and that have been shown to be stably associated with subsequent internalizing problems, taking into account questionnaire length and respondent compliance in a large-sample survey. Previous studies have shown that physical abuse, sexual abuse, emotional abuse, and neglect/caregiving deprivation are all associated with increased risks of subsequent internalizing problems such as depression and anxiety. Therefore, although the “childhood harmful exposure” indicator constructed in this study is not equivalent to the total score of the standard CTQ-SF, it has a clear empirical basis for representing distal adversity exposure related to emotional risk among university students (73). All items were scored on a four-point scale: 0 = “never, “ 1 = “occasionally, “ 2 = “often, “ and 3 = “almost every day, “ with higher scores indicating a higher frequency or greater severity of the corresponding harmful exposure. Because the seven items used in this study were all scored in the same positive direction (0–3), the item scores were directly summed after directional consistency processing to form a composite indicator of “childhood harmful exposure.” The total score ranged from 0 to 21, with higher scores indicating greater levels of harmful exposure experienced during childhood. In this study, this indicator was used to represent the level of distal adversity exposure within SPM framework. The internal consistency of this indicator in the present sample was good (Cronbach’s α = 0.809).

Family interaction in this study comprised family functioning, family communication, and family conflict, and was used to characterize the quality of the family relational context and its interaction patterns among university students.Family functioning was measured using the Chinese version of the Family Adaptability and Cohesion Evaluation Scales II (FACES II-CV). The original FACES II was developed by Olson et al. (74, 75), and the Chinese version was revised by Fei et al. (76). It is primarily used to assess two core dimensions of family systems: cohesion and adaptability. The scale contains 30 items in total, including 16 items measuring family cohesion and 14 items measuring family adaptability. Each item is rated on a five-point Likert scale, ranging from 1 (“almost never”) to 5 (“almost always”). After directional consistency processing in accordance with the scoring requirements of the scale, the scores of items corresponding to “actual cohesion” and “actual adaptability” were summed to generate dimension scores. Higher scores indicate closer emotional bonding among family members and greater flexibility of the family system in adapting to environmental and developmental changes (75). In the present sample, both dimensions showed good internal consistency (actual cohesion α = 0.855; actual adaptability α = 0.875).Family communication was measured using the Parent–Adolescent Communication Scale (PACS). The PACS was developed by Barnes and Olson (77) to assess the quality of communication between parents and adolescents. It includes two dimensions: open communication and problems in communication. Open communication reflects the extent to which family members are able to express emotions openly, exchange information, and discuss problems, whereas problems in communication reflects negative interaction characteristics in communication, such as avoidance, misunderstanding, blame, or blocking. The scale consists of 20 items, with 10 items measuring open communication and 10 items measuring problems in communication. Items are rated on a five-point Likert scale. Following directional consistency processing according to the scoring rules of the scale, item scores within each dimension were summed to obtain the dimension scores. Higher scores indicate a higher level of open communication or a greater degree of problematic communication, respectively (78). In this study, the PACS was mainly used to characterize the patterns of information expression, emotional exchange, and problem discussion among family members. Both dimensions demonstrated high internal consistency in the present sample (open communication α = 0.940; problems in communication α = 0.913).Family conflict was not measured using a single standardized scale. Instead, a composite indicator was constructed based on three items in the questionnaire reflecting high-conflict family interactions: “family members blame each other, “ “family members often quarrel or even fight, “ and “people in the family often shout at each other.” The first item was scored using a five-point frequency scale (1 = almost never, 2 = rarely, 3 = sometimes, 4 = often, 5 = always), whereas the latter two items used binary scoring (1 = yes, 0 = no). Because the three items used different response formats and measurement scales, each item was standardized after directional alignment, and an equal-weight sum was used to construct the family conflict index, thereby improving comparability and interpretive consistency of the composite indicator. Higher scores on this index indicate more pronounced conflict, blame, and high-arousal tense interactions among family members. In this study, this indicator was used to characterize negative interaction patterns within the family relational context.

Social support was measured using the Social Support Rating Scale (SSRS). The SSRS was developed by Xiao Shuiyuan and is a widely used instrument for assessing social support in Chinese populations. It is primarily designed to evaluate the overall level of social support that individuals receive and can utilize. The scale consists of 10 items. Following the original scoring rules of the scale, each item was assigned a score, and the scores of all 10 items were summed to obtain the total score, with higher scores indicating higher levels of social support (79). In this study, the SSRS was used to characterize the level of psychosocial resources that university students could mobilize and was included in the analysis as a resource pathway variable (80). The scale demonstrated good internal consistency in the present sample (Cronbach’s α = 0.854).

Within the domain of proximal functional status, this study focused on sleep status. Sleep status was not measured using the total score of a single standardized scale; instead, a composite indicator was constructed based on core symptom dimensions commonly used in previous sleep research and epidemiological surveys, combined with the structure of the present questionnaire. Prior studies have commonly regarded difficulty initiating sleep, difficulty maintaining sleep, insufficient sleep duration, and impaired daytime functioning as key symptom dimensions for characterizing sleep status (81, 82). Accordingly, the following four items were used to represent sleep status in this study:

1. Sleep latency: “During the past month, how long did it usually take you to fall asleep?” (<15 minutes = 0; 16–30 minutes = 1; 31–60 minutes = 2; >60 minutes = 3);
2. Sleep duration: “Approximately how many hours did you actually sleep per night?” (>7 hours = 0; 6–7 hours = 1; 5–6 hours = 2; <5 hours = 3);
3. Nighttime awakenings: “Do you wake easily during the night?” (No = 0; Yes = 1);
4. Daytime sleepiness: “Does daytime sleepiness affect your work or study?” (No = 0; 1–2 times per week = 1; ≥3 times per week = 2; every day = 3).

Because the response formats and scales of the items differed, each item was standardized after directional alignment, and an equal-weight sum was used to construct the sleep status composite index. Higher scores on this index indicate more severe sleep problems. In the present study, sleep status was primarily used to represent proximal functional status within the risk structure of anxiety and depression among university students.

Recent major negative life events (yes/no) were included to indicate whether individuals had recently been exposed to acute real-life stressors, thereby reflecting their recent stress burden. Lifestyle-related behaviors included average daily screen time, regular physical exercise, caffeine intake, alcohol consumption, and smoking status. These variables were included to characterize modifiable behavioral background factors and to reflect individuals’ behavioral exposure related to circadian rhythms, self-regulation, and physical condition. The coding of categorical variables is presented in **Table 1**.

### 2.3 Statistical analysis

After completing data cleaning according to the procedures described in Section 2.1 (Study design and participants), statistical analyses were conducted on all included samples. Categorical variables were described using frequencies and proportions n(%), and continuous variables were described using the mean ± standard deviation ( $\bar{x}\pm s$).Subsequently, group comparisons were conducted using the presence or absence of depressive state and anxiety state as grouping variables. For categorical variables, between-group comparisons were performed using the Pearson χ^2^ test. All continuous variables deviated from a normal distribution according to normality tests; however, given the large sample size in this study and the comprehensive assessment of variable distributions using histograms and Q–Q plots, the continuous variables exhibited only mild skewness. According to the central limit theorem, the distribution of sample means can therefore be approximated as normal. In addition, methodological studies have shown that the t-test and other parametric methods are relatively robust to mild departures from normality (83). Accordingly, independent-samples t-tests were used for comparisons of continuous variables between groups. Levene’s test was simultaneously applied to assess homogeneity of variance: when the assumption of equal variance was satisfied, the “equal variances assumed” results were reported; when the assumption was violated, the “equal variances not assumed” results were reported, corresponding to the Welch-corrected t-test. All tests were two-sided, with *P* < 0.05 considered statistically significant.

To explore the associations between factors at different levels and depressive state or anxiety state, and to examine changes in model explanatory power as variables were progressively introduced, regression models were constructed with depressive state and anxiety state as outcome variables. Because these outcomes were binary variables, binary logistic regression was used for analysis. To ensure the stability of model estimation, multicollinearity diagnostics were conducted for all candidate independent variables before model construction by calculating Tolerance and the Variance Inflation Factor (VIF). Generally, a Tolerance < 0.1 or VIF > 10 indicates substantial multicollinearity (84). Variables meeting these criteria were excluded from the final regression model.

Because the aim of this study was to examine the associations between distal-to-proximal factors and depressive/anxiety states, hierarchical binary logistic regression was applied. Depressive state and anxiety state were treated as dependent variables, respectively, and variables were entered stepwise according to the following order: demographic and baseline background → distal adversity exposure (childhood harmful exposure) → family interaction (FACES cohesion, adaptability; PACS open communication, problems in communication; family conflict) → social support → proximal stress/behavior (recent major negative life events, average daily screen time, regular exercise, caffeine intake, alcohol consumption, and smoking) → sleep status.Following this procedure, six hierarchical models were constructed for each outcome (depressive state and anxiety state). Regression results were presented as odds ratios (ORs) with 95% confidence intervals (95% CIs). Model fit and the incremental explanatory power contributed by each block of variables were evaluated using changes in −2 Log Likelihood, likelihood ratio tests, and Nagelkerke R (85, 86).

To further evaluate the predictive contribution of different factors to the classification of depression/anxiety risk and to compare the performance of different algorithms in this binary classification task, machine learning classification models were constructed with depressive state and anxiety state as outcome variables. Considering that the data in this study consisted of structured phenotypic information and that potential predictors may involve nonlinear relationships and higher-order interactions, three representative supervised learning algorithms were selected for comparison: Support Vector Machine (SVM), a margin-maximization discriminative model (87); Multilayer Perceptron (MLP), a neural network model capable of fitting complex nonlinear relationships; and LightGBM, a gradient boosting tree model suitable for tabular data and capable of capturing nonlinearities and variable interactions (88, 89). Comparing algorithms from different model families helps reduce the contingency associated with selecting a single model and improves the robustness of the results.

Because the number of positive samples for depression/anxiety was substantially smaller than that of negative samples, class weighting was applied to assign a higher penalty weight to the minority class in order to mitigate class imbalance while retaining all original sample information. Model training and evaluation were conducted using stratified five-fold cross-validation to ensure that the proportion of positive and negative samples in each fold remained approximately consistent with the overall distribution, thereby reducing random error and the risk of overfitting associated with a single data split.

For model performance evaluation, accuracy, precision, recall, F1 score, and the area under the receiver operating characteristic curve (AUC) were jointly reported. The mean values obtained from five-fold cross-validation were used to compare overall model performance and to identify the optimal model (90). Among these metrics, precision, recall, and the F1 score were used to more fully reflect the model’s ability to identify positive cases, whereas AUC was used to assess the overall discriminative performance of the model (91).

To enhance the interpretability of the final model, SHapley Additive exPlanations (SHAP) were further applied for model interpretation after identifying the optimal model. A SHAP summary plot was used to present the global ranking of feature importance and the overall direction of influence of feature values on model output. In addition, SHAP dependence plots were used to further illustrate the marginal contribution and variation trends of key variables to the predicted risk of depression/anxiety across different value levels, thereby improving the interpretability and clinical readability of the model results (92).

Finally, mediation analysis was conducted to further decompose the association effects between variables that were identified as stable correlates or important predictors in both hierarchical regression and machine learning analyses and the outcomes of depressive state and anxiety state, and to compare their indirect association patterns across the two outcomes. Given that the present study employed a cross-sectional design, the mediation analysis results were used only to describe the decomposition structure of association effects and were not interpreted as confirming causal temporal relationships. In the mediation models, *Y* represented the fixed outcome variable (depressive state or anxiety state), *X* represented the candidate antecedent variable, and *M* represented the candidate mediator. The association effect was decomposed into the total effect, direct effect, and indirect effect; in models with multiple mediators, specific indirect effects were further estimated. Effects were expressed as regression coefficients *B* (log-odds), with standard errors (SE) and 95% confidence intervals (CI) reported. Indirect effects were tested using the bootstrap method, and an indirect effect was considered statistically significant when its 95% CI did not include 0 (93). For variables measured within the same time frame whose temporal order was less clearly defined, reverse models were additionally constructed as sensitivity analyses. For variables with a clearly prior temporal attribute, such as childhood harmful exposure, reverse paths were not specified.

### 2.4 Ethical considerations

This study was conducted in strict accordance with the Declaration of Helsinki and relevant ethical guidelines. Questionnaires were collected anonymously via an online platform, and no information that could directly identify participants was obtained. All participants reviewed the electronic informed consent form and voluntarily provided consent after being informed of the study objectives, procedures, and data use. All data were analyzed and reported in anonymized form and stored on password-protected devices accessible only to authorized members of the research team. Study findings are used solely for academic publication and scientific dissemination, with participant privacy and data security fully protected. The study protocol was submitted to the Institutional Review Board (IRB) of the First Affiliated Hospital of Heilongjiang University of Chinese Medicine and was determined to be exempt from ethical review.

## 3 Results

### 3.1 Participant characteristics

A total of 10, 841 university students completed the online questionnaire. After excluding invalid responses, 9, 796 question naires were retained, yielding an effective response rate of 90.36% (9, 796/10, 841). Among the participants, 6, 154 were female (62.8%). A total of 1, 932 participants (19.7%) met the criteria for depressive status, and 1, 907 (19.5%) met the criteria for anxiety status. Baseline characteristics of the participants and between-group differences by depression/anxiety status are presented in **Table 2**.

**Table 2:** Participant characteristics and group comparisons by depression and anxiety status.

	Depressive status			Anxiety status		
Characteristics	No(n=7864)	Yes(n=1932)	*P* value	No(n=7889)	Yes(n=1907)	*P* value
Participant characteristics, n (%)						
**Sex**			0.002			0.057
Male	2866 (36.4)	776 (40.2)		2897 (36.7)	745 (39.1)	
Female	4998 (63.6)	1156 (59.8)		4992 (63.3)	1162 (60.9)	
**History of chronic physical disease**			<0.001			<0.001
NO	7758 (98.7)	1837 (95.1)		7782 (98.6)	1813 (95.1)	
YES	106 (1.3)	95 (4.9)		107 (1.4)	94 (4.9)	
Family characteristics, n (%)						
**Only-child status**			<0.001			<0.001
YES	3716 (47.3)	798 (41.3)		3718 (47.1)	796 (41.7)	
NO	4148 (52.7)	1134 (58.7)		4171 (52.9)	1111 (58.3)	
**Living arrangement with parents**			<0.001			<0.001
Living together	4841 (61.6)	1101 (57.0)		4845 (61.4)	1097 (57.5)	
Not living together but meeting weekly	804 (10.2)	161 (8.3)		801 (10.2)	164 (8.6)	
Not living together and meeting less than once per month	2219 (28.2)	670 (34.7)		2243 (28.4)	646 (33.9)	
**Parental marital status**			<0.001			<0.001
Married	6837 (86.9)	1589 (82.2)		6838 (86.7)	1588 (83.3)	
Divorced/separated	833 (10.6)	281 (14.5)		866 (10.8)	259 (13.6)	
Widowed	194 (2.5)	62 (3.2)		196 (2.5)	60 (3.1)	
**Father’s highest education level**			0.087			0.144
Junior high school or below	3733 (47.5)	974 (50.4)		3747 (47.5)	960 (50.3)	
Senior high school/technical secondary school	2727 (34.7)	641 (33.2)		2733 (34.6)	635 (33.3)	
Bachelor’s degree	1278 (16.3)	294 (15.2)		1286 (16.3)	286 (15.0)	
Master’s degree or above	126 (1.6)	23 (1.2)		123 (1.6)	26 (1.4)	
**Mother’s highest educational level**			0.029			0.051
Junior high school or below	4063 (51.7)	1064 (55.1)		4076 (51.7)	1051 (55.1)	
Senior high school/technical secondary school	2572 (32.7)	569 (29.5)		2561 (32.5)	580 (30.4)	
Bachelor’s degree	1144 (14.5)	275 (14.2)		1165 (14.8)	254 (13.3)	
Master’s degree or above	85 (1.1)	24 (1.2)		87 (1.1)	22 (1.2)	
**Father’s occupational category**			0.394			0.807
Enterprise employee	1136 (14.4)	273 (14.1)		1141 (14.5)	268 (14.1)	
Civil servant/Public institution staff	1088 (13.8)	252 (13.0)		1087 (13.8)	253 (13.3)	
Self-employed	2168 (27.6)	506 (26.2)		2163 (27.4)	511 (26.8)	
Agriculture/Migrant labor	2682 (34.1)	699 (36.2)		2701 (34.2)	680 (35.7)	
Other	790 (10.0)	202 (10.5)		797 (10.1)	195 (10.2)	
**Mother’s occupational category**			0.438			0.366
Enterprise employee	984 (12.5)	241 (12.5)		981 (12.4)	244 (12.8)	
Civil servant/Public institution staff	991 (12.6)	216 (11.2)		990 (12.5)	217 (11.4)	
Self-employed	2169 (27.6)	527 (27.3)		2188 (27.7)	508 (26.6)	
Agriculture/Migrant labor	2522 (32.1)	649 (33.6)		2524 (32.0)	647 (33.9)	
Other	1198 (15.2)	299 (15.5)		1206 (15.3)	291 (15.3)	
**Family economic status**			<0.001			<0.001
Financial difficulty	1143 (14.5)	426 (22.0)		1172 (14.9)	397 (20.8)	
Average	5920 (75.3)	1380 (71.4)		5919 (75.0)	1381 (72.4)	
Affluent	801 (10.2)	126 (6.5)		798 (10.1)	129 (6.8)	
**Family history of mental disorders (first-degree relatives)**			<0.001			<0.001
No	7769 (98.8)	1843 (95.4)		7784 (98.7)	1828 (95.9)	
Yes	95 (1.2)	89 (4.6)		105 (1.3)	79 (4.1)	
Health and lifestyle characteristics, n (%)						
**Average daily screen time**			<0.001			<0.001
<2h	640 (8.1)	46 (2.4)		642 (8.1)	44 (2.3)	
2-4h	2001 (25.4)	337 (17.4)		1991 (25.2)	347 (18.2)	
4-6h	2583 (32.8)	608 (31.5)		2601 (33.0)	590 (30.9)	
6-8h	1709 (21.7)	497 (25.7)		1691 (21.4)	515 (27.0)	
8-10h	614 (7.8)	252 (13.0)		634 (8.0)	232 (12.2)	
10-12h	189 (2.4)	103 (5.3)		195 (2.5)	97 (5.1)	
>12h	128 (1.6)	89 (4.6)		135 (1.7)	82 (4.3)	
**Regular physical activity**			<0.001			<0.001
≥ 3 times/week	2979 (37.9)	518 (26.8)		2975 (37.7)	522 (27.4)	
1–2 times/week	3902 (49.6)	944 (48.9)		3904 (49.5)	942 (49.4)	
Rarely	983 (12.5)	470 (24.3)		1010 (12.8)	443 (23.2)	
**Caffeine intake**			<0.001			<0.001
≥ 1 cup/day	396 (5.0)	175 (9.1)		404 (5.1)	167 (8.8)	
Occasional	4516 (57.4)	1123 (58.1)		4509 (57.2)	1130 (59.3)	
None	2952 (37.5)	634 (32.8)		2976 (37.7)	610 (32.0)	
**Alcohol consumption**			<0.001			<0.001
Never	4966 (63.1)	966 (50.0)		4941 (62.6)	991 (52.0)	
Occasional	2876 (36.6)	950 (49.2)		2920 (37.0)	906 (47.5)	
Daily	22 (0.3)	16 (0.8)		28 (0.4)	10 (0.5)	
**Smoking status**			<0.001			<0.001
Never	7127 (90.6)	1624 (84.1)		7121 (90.3)	1630 (85.5)	
Occasional	525 (6.7)	184 (9.5)		534 (6.8)	175 (9.2)	
Daily	212 (2.7)	124 (6.4)		234 (3.0)	102 (5.3)	
Current status						
**Recent major negative life events**			<0.001			<0.001
No	6546 (83.2)	1273 (65.9)		6551 (83.0)	1268 (66.5)	
Yes	1318 (16.8)	659 (34.1)		1338 (17.0)	639 (33.5)	
**Sleep status, mean ± SD**	-0.59 ± 2.12	2.39 ± 2.76	<0.001	-0.52 ± 2.17	2.14 ± 2.88	<0.001
Family interaction						
**Actual cohesion, mean ± SD**	75.90 ± 10.39	66.79 ± 10.89	<0.001	75.73 ± 10.49	67.37 ± 11.00	<0.001
**Actual adaptability, mean ± SD**	55.41 ± 9.34	47.36 ± 9.93	<0.001	55.29 ± 9.45	47.78 ± 9.90	<0.001
**Open communication, mean ± SD**	40.91 ± 7.53	34.53 ± 7.79	<0.001	40.83 ± 7.57	34.76 ± 7.82	<0.001
**Problematic communication, mean ± SD**	21.72 ± 8.04	28.53 ± 6.77	<0.001	21.81 ± 8.09	28.23 ± 6.85	<0.001
**Family conflict, mean ± SD**	-0.37 ± 1.83	1.52 ± 3.22	<0.001	-0.34 ± 1.88	1.42 ± 3.18	<0.001
**Social support, mean ± SD**	29.00 ± 5.13	24.98 ± 4.55	<0.001	28.95 ± 5.16	25.13 ± 4.52	<0.001
**Childhood harmful exposure, mean ± SD**	5.83 ± 1.37	7.44 ± 2.40	<0.001	5.85 ± 1.40	7.39 ± 2.39	<0.001
**Age, mean ± SD**	19.26 ± 1.88	19.22 ± 1.82	0.380	19.28 ± 1.88	19.17 ± 1.80	0.031

Categorical variables are presented as *n*(%) and were compared using the Pearson χ2 test; continuous variables are presented as $\bar{x}\pm s$ and were compared using the independent-samples t test, with Welch’s correction applied when the assumption of equal variances was violated. Bold P values indicate statistically significant between-group differences (P<0.05).

Both the depression and anxiety groups showed higher proportions of chronic physical diseases and a family history of mental disorders among first-degree relatives compared with their respective non-depression/non-anxiety groups. Non-married family structures (e.g., divorced, separated, or widowed parents) and poorer family economic status were also more common in the depression/anxiety groups. Regarding health and lifestyle factors, consistent patterns were observed: longer average daily screen time, less regular physical activity, and higher prevalence of caffeine intake, alcohol consumption, and smoking were more concentrated in the depression/anxiety groups. In addition, recent major negative life events were more frequently reported among participants with depression or anxiety.

In terms of family functioning, participants in the depression/anxiety groups were more likely to be classified as cohesive in cohesion and structured in adaptability, whereas those without depression/anxiety more often exhibited enmeshed or flexible patterns; overall, the depression/anxiety groups tended toward more unbalanced family types. With respect to parent–child communication, the depression/anxiety groups were characterized by lower levels of open communication and more prominent problem communication, accompanied by poorer social support, worse sleep status, and higher levels of family conflict.

### 3.2 Multicollinearity diagnostics

The results of the multicollinearity diagnostics are presented in **Table 3**. All variables had tolerance values greater than 0.10 and variance inflation factors (VIFs) below 10, indicating no evidence of severe multicollinearity in the model. Variables with relatively higher VIF values were mainly related to family interaction indicators, with actual cohesion and actual adaptability showing the highest values, followed by open communication. This suggests that these conceptually related variables share some variance, but the level of overlap did not reach a degree that would compromise the stability of model estimation. VIF values for social support, family conflict, and other background and behavioral variables were generally low. Therefore, all variables were retained and included in the subsequent hierarchical logistic regression analyses.

**Table 3:** Multicollinearity diagnostics.

Variable	Tolerance	VIF	Variable	Tolerance	VIF
Sex	0.779	1.284	Regular physical activity	0.819	1.221
Age	0.892	1.121	Caffeine intake	0.916	1.092
History of chronic physical disease	0.958	1.044	Alcohol consumption	0.804	1.244
only-child status	0.865	1.156	Smoking status	0.810	1.234
Living arrangement with parents	0.959	1.043	Recent major negative life events	0.928	1.077
Parental marital status	0.946	1.057	Sleep status	0.755	1.325
Father’s highest education level	0.498	2.006	Actual cohesion	0.168	5.937
Mother’s highest educational level	0.473	2.115	Actual adaptability	0.191	5.236
Father’s occupational category	0.576	1.735	Open communication	0.291	3.435
Mother’s occupational category	0.586	1.706	Problematic communication	0.480	2.083
Family economic status	0.813	1.230	Family conflict	0.594	1.683
Family history of mental disorders (first-degree relatives)	0.947	1.055	Social support	0.551	1.815
Average daily screen time	0.878	1.139	Childhood harmful exposure	0.606	1.649

### 3.3 Hierarchical binary logistic regression

Hierarchical binary logistic regression analyses were conducted to further examine the university student data in this study. Variables were entered stepwise in the order of “ baseline background→childhood harmful exposure→family interaction→social support→proximal stress and behavioral exposures→sleep status”, and six nested models were constructed separately for depressive status and anxiety status. Overall, model fit improved significantly for both outcomes as variables were added. Specifically, with stepwise inclusion, −2 Log Likelihood decreased continuously (depression: 9451.277→ 6801.895^a^), Omnibus tests remained significant, and Nagelkerke *R*2 increased in a stepwise manner (depression: 0.044→0.410; anxiety: 0.035→0.348). These results indicate that variables at different levels provided additional statistical information for both outcomes; however, incremental contributions across levels and the stability of key variables differed.

After inclusion of demographic and baseline background variables (Model 1), both the depressive and anxiety models reached overall statistical significance; however, the explanatory power was limited (Nagelkerke *R*2: 0.044 for depression; 0.035 for anxiety). This indicates that demographic and baseline background factors have relatively weak explanatory capacity for depression and anxiety and are more likely to reflect baseline differences. Notably, sex was significantly associated with depression only, suggesting that depressive status may be more sensitive to sex differences than anxiety, although the overall effect size remained small.

After adding childhood harmful exposure to Model 1 (Model 2), the explanatory power of both models increased markedly (Nagelkerke *R*2: 0.185 for depression; 0.166 for anxiety), indicating that childhood experiences contribute substantially more to explaining internalizing symptoms than demographic and baseline factors. childhood harmful exposure were positively associated with both depression and anxiety (depression OR = 1.540; anxiety OR = 1.509) and remained independently associated after adjustment for demographic and baseline background variables. Concurrently, the effects of some family structure variables observed in Model 1 were attenuated, suggesting that childhood experiences may partially account for the association between family structure and psychological outcomes. Compared with the anxiety model, childhood harmful exposure showed greater explanatory relevance for depression (depression OR = 1.540 vs. anxiety OR = 1.509).

In Model 3, family interaction —FACES cohesion and adaptability, PACS open communication and problem communication, and family conflict—were added to Model 2. Overall model performance improved substantially for both depression and anxiety; however, the magnitude of improvement was greater for the depression model (Nagelkerke *R*2: depression 0.185 → 0.260; anxiety 0.166 → 0.231).At the variable level, only-child status showed independent associations with both depression and anxiety in Model 2, but became completely non-significant after the inclusion of family interaction variables, indicating that the association between only-child status and psychological outcomes is unlikely to be direct and is more plausibly mediated by family interaction. childhood harmful exposure remained robust after adding family interaction, although the ORs were attenuated, suggesting partial mediation through the quality of recent family interaction. Notably, problem communication and family conflict emerged as the most influential factors in Model 3, contributing substantially more to model explanatory power than structural background variables; positive family functioning also remained significant. Age and history of chronic physical disease remained significant in both the depression and anxiety models across strata.Comparing the two outcomes, family economic status and family history of mental disorders among first-degree relatives lost significance in the anxiety model but remained significant in the depression model, indicating that these factors are more readily explained by recent family interaction quality in anxiety. In addition, family cohesion was significant only in the depression model, suggesting that depression may be more sensitive to emotional support within the family.

In Model 4, the addition of social support led to consistent improvements in both models (Nagelkerke *R*2: depression 0.260 → 0.276; anxiety 0.231 → 0.248), with comparable magnitudes of increase. In both Model 4 specifications, social support showed a strong independent effect (OR = 0.927 for both depression and anxiety).At the variable level, indicators of positive family functioning lost statistical significance in the depression model, whereas open communication in the anxiety model, although attenuated, remained significant. This pattern suggests that the protective effects of positive family functioning on depression may operate largely through individuals’ accessible social support, while anxiety appears to be more directly sensitive to communication styles. In contrast, negative family interactions (problem communication and family conflict) were not offset by social support in either model, indicating that negative family interaction and social support may represent two parallel pathways in the development of depression and anxiety. Additionally, the effects of childhood harmful exposure showed minimal attenuation in Model 4, underscoring the continued importance of early-life risk within models that account for recent background factors, family influences, and social support.

In Model 5, further inclusion of proximal behaviors and events—major negative life events, average daily screen time, regular physical activity, caffeine intake, alcohol consumption, and smoking—led to additional increases in explanatory power for both outcomes (Nagelkerke *R*2: depression 0.276 → 0.304; anxiety 0.248 → 0.271), indicating that negative life events and lifestyle factors provided incremental explanatory value.At the variable level, social support (depression OR = 0.932; anxiety OR = 0.931), childhood harmful exposure (depression OR = 1.227; anxiety OR = 1.221), problem communication (depression OR = 1.043; anxiety OR = 1.039), and family conflict (depression OR = 1.068; anxiety OR = 1.058) remained significant risk factors with comparable effect sizes across the two models. Open communication (OR = 0.598) remained significant in the anxiety model. Among the newly added variables, major negative life events, average daily screen time, regular physical activity, and caffeine intake were all significant, with major negative life events showing particularly strong associations (depression OR = 1.643; anxiety OR = 1.645), followed by average daily screen time. Smoking (P<0.001) and alcohol consumption (P = 0.006) were significantly associated with depression but were not significant in the anxiety model.

In Model 6, sleep status was added to Model 5, resulting in further improvements in both explanatory power and discriminative ability for the two outcomes (Nagelkerke *R*2: depression increased from 0.304 to 0.410; anxiety from 0.271 to 0.348), as shown in **Table 4**. This indicates that, after extensive adjustment for preceding variables, the inclusion of sleep status provided additional explanatory value, with sleep status showing a significant effect in both models (depression OR = 1.431; anxiety OR = 1.335).In Model 6, social support, childhood harmful exposure, problem communication, family conflict, major negative life events, average daily screen time, and regular physical activity exhibited stable and significant associations with both depression and anxiety. In contrast, chronic physical disease and open communication reached statistical significance only in the anxiety model, whereas daily smoking was significantly associated with depression only.

**Table 4:** Hierarchical binary logistic regression models (model 6).

Variable	Depressive status				Anxiety status			
	B	*P*	OR	95% CI for OR	B	*P*	OR	95% CI for OR
**Sex(1=male, 2=female)**	0.018	0.798	1.019	(0.885, 1.173)	0.056	0.417	1.058	(0.923, 1.213)
**Age(years)**	-0.082	0.000	0.921	(0.888, 0.955)	-0.092	0.000	0.913	(0.881, 0.945)
**Living arrangement with parents**		0.695				0.607		
Living together	ref	ref	ref	ref	ref	ref	ref	ref
Not living together but meeting weekly	-0.068	0.551	0.934	(0.747, 1.168)	-0.010	0.929	0.990	(0.800, 1.226)
Not living together and meeting < once per month	-0.050	0.469	0.951	(0.830, 1.089)	-0.067	0.322	0.936	(0.820, 1.067)
**Only-child status(1= No, 2= Yes)**	-0.077	0.261	0.926	(0.810, 1.059)	-0.042	0.525	0.959	(0.842, 1.091)
**Parental marital status**		0.625				0.140		
Married	ref	ref	ref	ref	ref	ref	ref	ref
Divorced/Separated	-0.089	0.360	0.915	(0.757, 1.106)	-0.187	0.050	0.830	(0.689, 1.000)
Widowed	0.044	0.814	1.045	(0.726, 1.503)	0.015	0.936	1.015	(0.712, 1.446)
**Father’s highest educational level**		0.117				0.634		
Junior high school or below	ref	ref	ref	ref	ref	ref	ref	ref
Senior high school/technical secondary school	0.062	0.438	1.064	(0.909, 1.246)	0.046	0.551	1.047	(0.900, 1.219)
Bachelor’s degree	-0.163	0.202	0.850	(0.662, 1.091)	-0.092	0.454	0.912	(0.716, 1.161)
Master’s degree or above	-0.540	0.110	0.583	(0.301, 1.130)	0.012	0.969	1.012	(0.556, 1.841)
**Mother’s highest educational level**		0.077				0.951		
Junior high school or below	ref	ref	ref	ref	ref	ref	ref	ref
Senior high school/technical secondary school	-0.036	0.670	0.965	(0.818, 1.138)	-0.029	0.721	0.972	(0.829, 1.139)
Bachelor’s degree	0.240	0.071	1.272	(0.979, 1.651)	-0.049	0.708	0.952	(0.738, 1.230)
Master’s degree or above	0.595	0.096	1.813	(0.899, 3.655)	0.091	0.792	1.095	(0.559, 2.145)
**Father’s occupational category**		0.251				0.330		
Enterprise employee	ref	ref	ref	ref	ref	ref	ref	ref
Civil servant/Public institution staff	0.066	0.633	1.068	(0.816, 1.398)	0.120	0.367	1.127	(0.869, 1.464)
Self-employed	-0.167	0.158	0.846	(0.671, 1.067)	-0.004	0.970	0.996	(0.796, 1.245)
Agriculture/Migrant labor	-0.069	0.619	0.934	(0.713, 1.223)	-0.145	0.278	0.865	(0.666, 1.124)
Other	-0.219	0.143	0.804	(0.600, 1.077)	-0.179	0.215	0.836	(0.629, 1.110)
**Mother’s occupational category**		0.321				0.762		
Enterprise employee	ref	ref	ref	ref	ref	ref	ref	ref
Civil servant/Public institution staff	-0.109	0.467	0.897	(0.669, 1.202)	-0.107	0.456	0.899	(0.678, 1.191)
Self-employed	0.170	0.163	1.185	(0.933, 1.505)	-0.051	0.666	0.951	(0.756, 1.196)
Agriculture/Migrant labor	0.029	0.840	1.029	(0.776, 1.366)	0.078	0.572	1.082	(0.824, 1.419)
Other	0.048	0.728	1.050	(0.799, 1.378)	-0.040	0.766	0.961	(0.740, 1.249)
**Family economic status**		0.472				0.689		
Financial difficulty	ref	ref	ref	ref	ref	ref	ref	ref
Average	-0.083	0.343	0.920	(0.775, 1.093)	-0.012	0.887	0.988	(0.835, 1.169)
Affluent	0.020	0.896	1.020	(0.761, 1.367)	0.090	0.531	1.095	(0.825, 1.452)
**Family history of mental disorders (first-degree relatives) (1= No, 2= Yes)**	0.149	0.445	1.161	(0.792, 1.702)	0.005	0.980	1.005	(0.690, 1.462)
**History of chronic physical disease (1= No, 2= Yes)**	0.329	0.087	1.390	(0.953, 2.026)	0.442	0.016	1.557	(1.087, 2.228)
**Childhood harmful exposure**	0.166	0.000	1.181	(1.137, 1.227)	0.166	0.000	1.180	(1.137, 1.224)
**Actual cohesion**	0.000	0.941	1.000	(0.988, 1.013)	0.009	0.169	1.009	(0.996, 1.021)
**Actual adaptability**	-0.008	0.257	0.992	(0.979, 1.006)	-0.008	0.199	0.992	(0.979, 1.004)
**Open communication**	-0.011	0.099	0.989	(0.976, 1.002)	-0.018	0.007	0.983	(0.970, 0.995)
**Problematic communication**	0.029	0.000	1.030	(1.019, 1.041)	0.027	0.000	1.028	(1.017, 1.038)
**family conflict**	0.064	0.000	1.066	(1.036, 1.096)	0.052	0.000	1.054	(1.025, 1.083)
**Social support**	-0.048	0.000	0.953	(0.939, 0.968)	-0.052	0.000	0.949	(0.936, 0.963)
**Recent major negative life events (1=NO, 2= Yes)**	0.405	0.000	1.499	(1.304, 1.724)	0.416	0.000	1.516	(1.325, 1.736)
**Average daily screen time**		0.001				0.000		
<2h	ref	ref	ref	ref	ref	ref	ref	ref
2-4h	0.553	0.004	1.738	(1.190, 2.539)	0.645	0.001	1.906	(1.317, 2.759)
4-6h	0.678	0.000	1.971	(1.363, 2.850)	0.714	0.000	2.042	(1.423, 2.931)
6-8h	0.709	0.000	2.031	(1.397, 2.953)	0.876	0.000	2.401	(1.666, 3.462)
8-10h	0.825	0.000	2.282	(1.534, 3.393)	0.820	0.000	2.270	(1.537, 3.351)
10-12h	0.943	0.000	2.568	(1.621, 4.070)	0.989	0.000	2.688	(1.713, 4.217)
>12h	0.843	0.001	2.324	(1.409, 3.834)	0.878	0.000	2.405	(1.476, 3.919)
**Regular physical activity**		0.000				0.016		
≥ 3 times/week	ref	ref	ref	ref	ref	ref	ref	ref
1–2 times/week	0.240	0.002	1.271	(1.095, 1.477)	0.178	0.015	1.195	(1.035, 1.380)
Rarely	0.366	0.000	1.443	(1.193, 1.744)	0.246	0.009	1.280	(1.064, 1.539)
**Caffeine intake**		0.429				0.028		
≥ 1 cup/day	ref	ref	ref	ref	ref	ref	ref	ref
Occasional	0.042	0.744	1.043	(0.810, 1.342)	0.027	0.826	1.028	(0.805, 1.312)
None	-0.048	0.724	0.953	(0.732, 1.242)	-0.151	0.247	0.859	(0.665, 1.111)
**Alcohol consumption**		0.061				0.461		
Never	ref	ref	ref	ref	ref	ref	ref	ref
Occasional	0.146	0.033	1.157	(1.012, 1.323)	0.054	0.418	1.055	(0.927, 1.202)
Daily	0.551	0.205	1.736	(0.740, 4.071)	-0.392	0.395	0.676	(0.274, 1.666)
**Smoking status**		0.051				0.766		
Never	ref	ref	ref	ref	ref	ref	ref	ref
Occasional	0.109	0.358	1.115	(0.884, 1.408)	0.075	0.518	1.078	(0.859, 1.353)
Daily	0.382	0.017	1.465	(1.071, 2.003)	0.071	0.654	1.074	(0.786, 1.468)
**sleep status**	0.359	0.000	1.431	(1.395, 1.469)	0.289	0.000	1.335	(1.303, 1.368)

Bold P values indicate statistically significant between-group differences (P<0.05).

With the stepwise inclusion of different categories of variables, the model fit for both depressive and anxiety states improved continuously. This suggests that childhood harmful exposure, negative family interaction and social resources, proximal stressors and behaviors, and sleep-related factors jointly constituted a stable framework of associations for both outcomes. Although the core associated variables were highly consistent between depression and anxiety, differences emerged in emphasis: anxiety showed stronger associations with individual health status and communication characteristics, whereas depression showed a more pronounced association with smoking behavior.

### 3.4 Machine learning models

**Figure 2:**
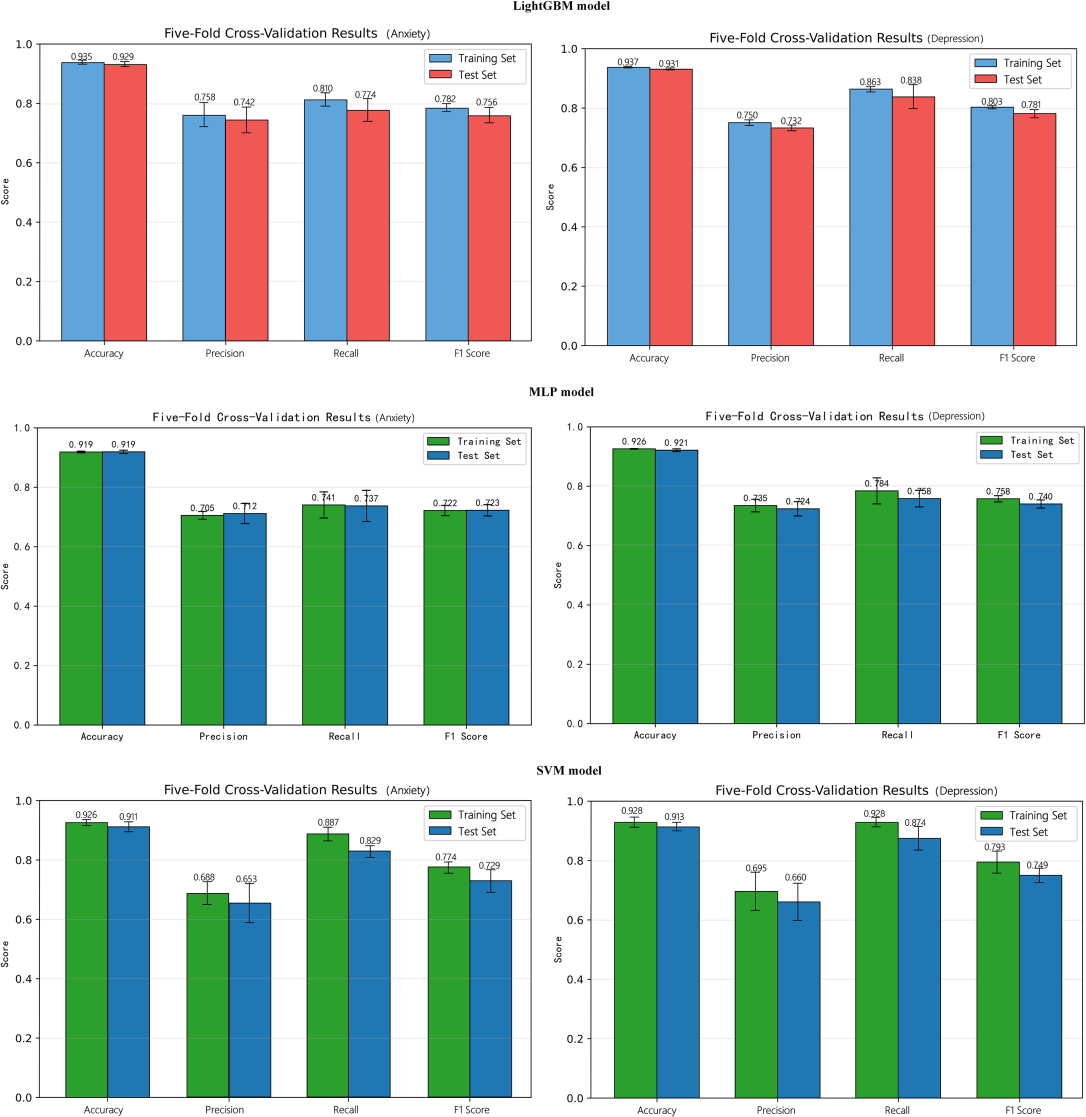
Grouped bar chart with error bars for the performance of LightGBM, MLP, and SVM models.

**Figure 3:**
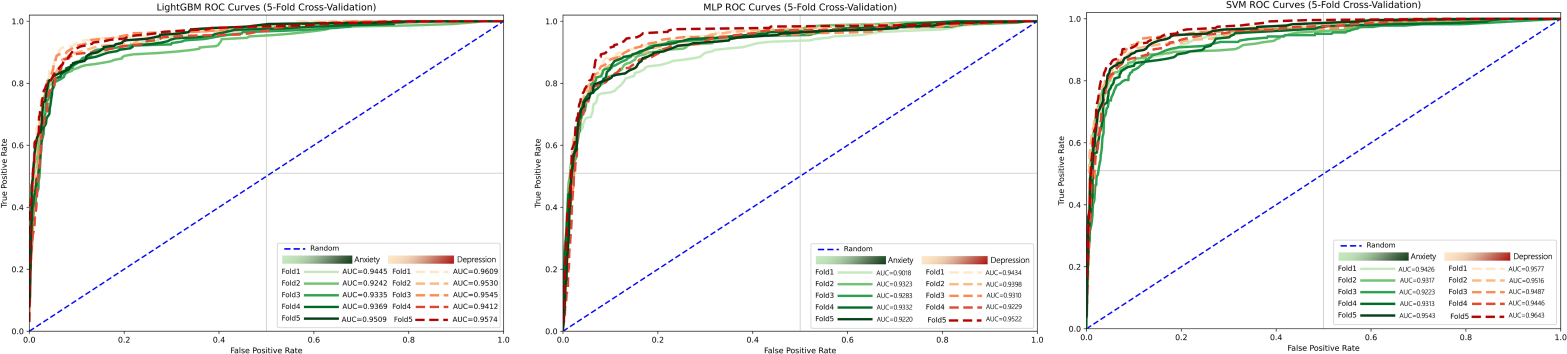
Five-fold cross-validation ROC curves for LightGBM, MLP, and SVM (Anxiety vs. Depression).

Comparison of the overall performance of the three models—LightGBM, MLP, and SVM—as shown in **Figures 2**, **3**, indicated that LightGBM demonstrated consistent advantages for both anxiety and depression. Specifically, the LightGBM model achieved test-set accuracy values of 0.929 for anxiety and 0.931 for depression. Its precision and F1 score were the highest among the three models (test-set precision: 0.742 for anxiety and 0.732 for depression; F1 score: 0.756 and 0.781, respectively), indicating fewer false-positive classifications and stronger overall discriminative performance in identifying anxiety and depression.

Across five-fold cross-validation, LightGBM consistently yielded high AUC values (anxiety: 0.924–0.951; depression: 0.941–0.961), suggesting greater stability and robustness in discrimination. Although the SVM model achieved higher recall, its precision and F1 scores were lower, resulting in inferior overall performance compared with LightGBM. Taken together, LightGBM was selected as the optimal model for subsequent analyses.

**Figure 4:**
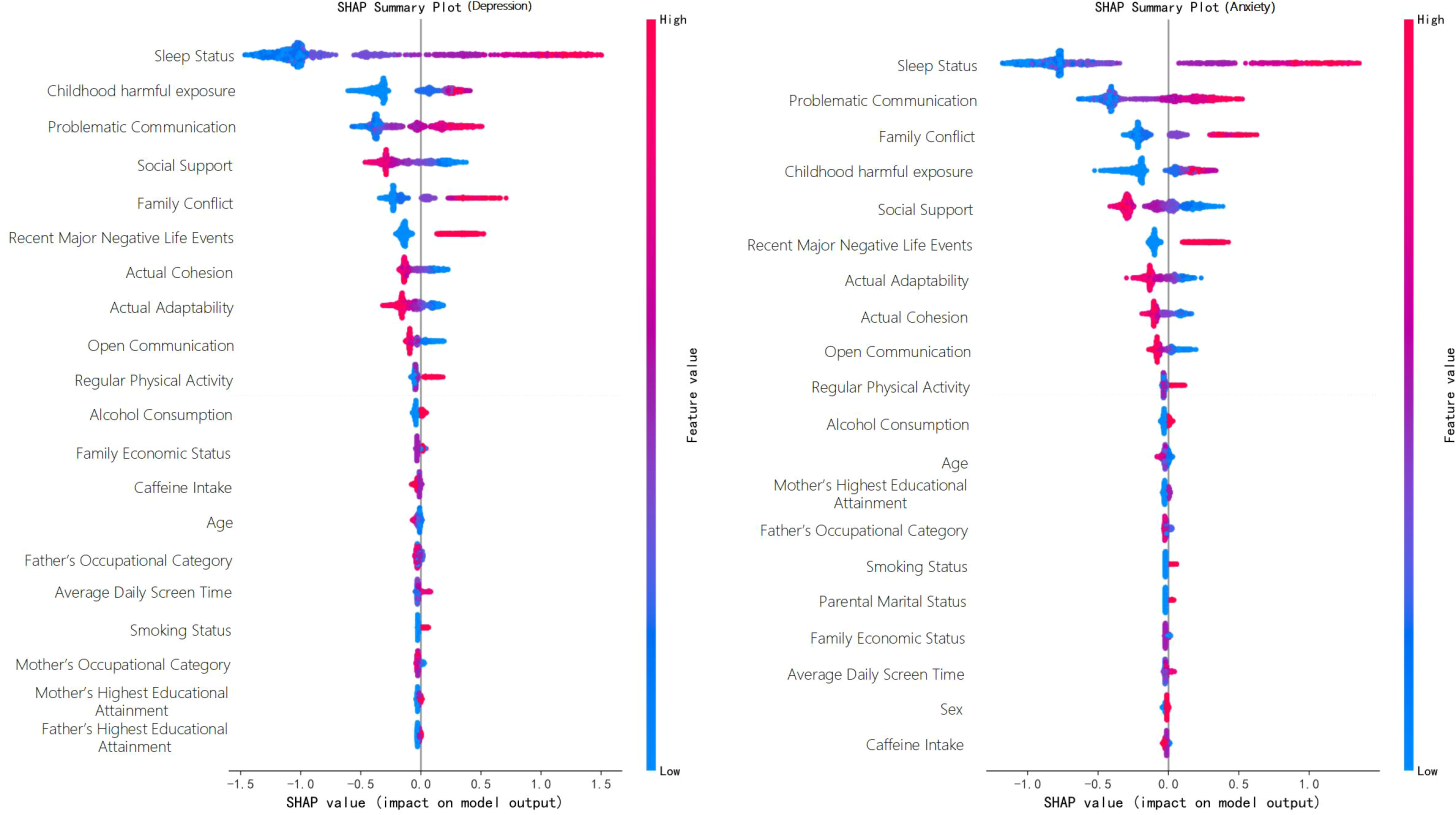
SHAP summary plots for the Anxiety and Depression models.

The optimal model (LightGBM) was further interpreted using SHAP summary plots (**Figure 4**). The results showed a high degree of consistency in the key contributing variables for both anxiety and depression. Sleep status exerted the strongest influence on the classification of anxiety/depression, with poorer sleep status corresponding to a higher probability of being classified as a positive outcome. childhood harmful exposure, problem communication, family conflict, and major negative life events all demonstrated stable positive contributions to the outcomes. Notably, childhood harmful exposure had a stronger impact on depressive status than on anxiety. Social support was characterized predominantly by negative SHAP values, indicating a protective effect. In contrast, most demographic variables and some behavioral factors exhibited SHAP values close to zero, suggesting limited overall contribution to model predictions.

**Figure 5:**
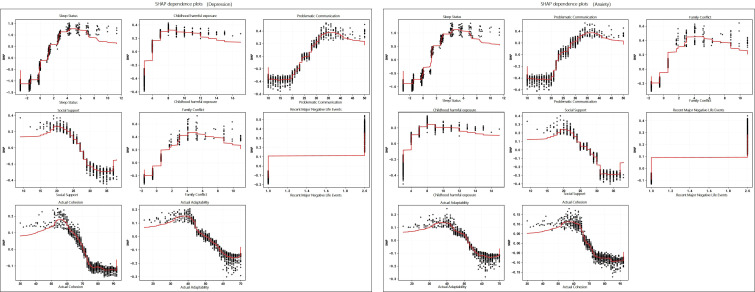
SHAP dependence plots of key predictors for depression and anxiety. The left panel presents SHAP dependence plots for depression, and the right panel presents those for anxiety. In each subplot, black dots represent individual samples and the red curve shows the overall dependence trend between the predictor and the SHAP value. These plots visualize the nonlinear associations of major predictors, including sleep status, childhood harmful exposure, problematic communication, social support, family conflict, recent major negative life events, actual cohesion, and actual adaptability, with the model output for depression and anxiety.

Combined SHAP dependence plots for the anxiety and depression models (**Figure 5**) revealed clear dose–response relationships between core variables and model outputs, with most relationships exhibiting nonlinear patterns. As sleep status worsened, SHAP values rapidly shifted from negative to positive and continued to increase, reaching peak values in the moderate-to-high range, indicating a markedly increased probability of positive anxiety/depression classification as sleep deteriorated. Problem communication showed a sharp increase in SHAP values beyond a score of 25, substantially elevating the likelihood of positive classification. Family conflict displayed a similar upward trend, suggesting that negative family interactions, once exceeding a certain level, substantially increase the probability of positive outcomes.For childhood harmful exposure, SHAP values were negative at low levels but transitioned from negative to positive and increased progressively as scores rose, indicating a threshold-like amplification of childhood harmful exposure to anxiety/depression. In contrast, social support showed a gradual decline in SHAP values with increasing scores, turning negative at approximately 25, suggesting that higher levels of social support (>25) can sustainably offset anxiety/depression risk. Comparing outcomes, the threshold at which SHAP values turned from positive to negative occurred at a slightly higher score in the depression model (≈27), implying that a higher level of social support may be required to reduce depressive status. The presence of recent major negative life events was associated with an overall upward shift in SHAP values, indicating a significant increase in anxiety/depression risk. Regarding family functioning, both actual cohesion and actual adaptability showed marked declines in SHAP values toward negative ranges at higher levels, suggesting stable protective effects of high family cohesion and adaptability against anxiety and depression.

Within family functioning, when actual cohesion increased to approximately 70, the risk of positive classification declined markedly; this range corresponds to a high cohesion level in the FACES II-CV (approaching the enmeshed type). Similarly, when actual adaptability increased to around 50, risk showed a clear downward trend, corresponding to the high adaptability range in the FACES II-CV (flexible–chaotic types) (94). These patterns indicate that high levels of family cohesion and adaptability exert stable protective effects against anxiety and depression.

Comparison of the SHAP results for anxiety and depression (**Figures 4**, **5**) showed that problem communication and family conflict were ranked higher in the anxiety model and exhibited steeper upward trends in the dependence plots. This indicates that anxiety is more sensitive to recent negative family interactions and conflict. In contrast, in the depression model, childhood harmful exposure ranked as the second most important contributor, and actual cohesion occupied a higher position. Together, these findings suggest that key contributors to anxiety are more concentrated in recent family interactions and conflict, whereas depression risk is more strongly related to earlier-life cumulative experiences.

Compared with the hierarchical binary logistic regression results, the SHAP summary plots for anxiety and depression showed largely consistent core variables. However, some variables that were statistically significant in the regression models—such as age and average daily screen time—exhibited relatively weak contributions in SHAP, whereas variables that were not significant in the regression models—such as actual cohesion/adaptability and open communication—demonstrated non-negligible contributions in SHAP. This discrepancy suggests that statistical significance and predictive importance are not equivalent. Hierarchical logistic regression reflects the independent association of variables after controlling for other covariates and is therefore more susceptible to shared variance among predictors and assumptions of linear model specification. In contrast, SHAP is based on nonlinear machine learning models and reflects the marginal contribution and ranking of variables within the overall prediction process, allowing it to capture nonlinear relationships, threshold effects, and interactions among variables more effectively. Consequently, although variables such as age and average daily screen time showed independent statistical associations with the outcomes, their incremental contribution to individual risk discrimination became relatively limited once stronger predictive signals were incorporated into the model. Conversely, although actual cohesion, actual adaptability, and open communication did not appear as stable independent effects in the regression models, their contribution within the machine learning models suggests that these positive dimensions of family functioning still contain discriminative information. In particular, they may improve the identification of anxiety and depressive states through joint effects with variables such as negative family interaction, social support, and sleep status. herefore, the results obtained from the two analytical approaches are not contradictory but rather reveal the statistical relationships between the predictors and outcomes from two complementary perspectives: independent association and overall prediction. This difference further indicates that the positive dimensions of family functioning may not constitute stable independent correlates after strict adjustment, but they may still provide supplementary value in risk identification. Their role is more likely reflected in jointly providing information on individual differences together with other psychosocial variables, rather than independently determining outcome status.

Overall, the SHAP analyses indicated that classification of both anxiety and depression was primarily driven by sleep status, early-life experiences, negative family interactions, social support, and recent major negative life events. However, differences were observed in the relative contributions and dose–response patterns of these factors between the two outcomes. These findings suggest that anxiety and depression share a largely overlapping core risk profile, while exhibiting subtle distinctions. Further research is warranted to more deeply investigate these between-outcome differences in risk patterns.

### 3.5 Mediation analysis

Based on findings from the hierarchical binary logistic regression and machine learning models, the key variables showing the most stable associations with anxiety and depressive states were identified as childhood harmful exposure, problematic communication/family conflict, social support, and sleep status. These variables consistently demonstrated stable statistical associations in the preceding analyses, suggesting that there may be underlying association patterns among them that warrant further decomposition.Accordingly, mediation analysis was conducted to estimate the total effect, direct effect, and indirect effect. The analysis examined whether the associations between childhood harmful exposure and negative family interactions with depressive/anxiety states could be partially accounted for by indirect effects through social support and sleep status, and compared the magnitude of indirect effects across different antecedent variables and different outcomes.

#### 3.5.1 Main analysis

**Figure 6:**
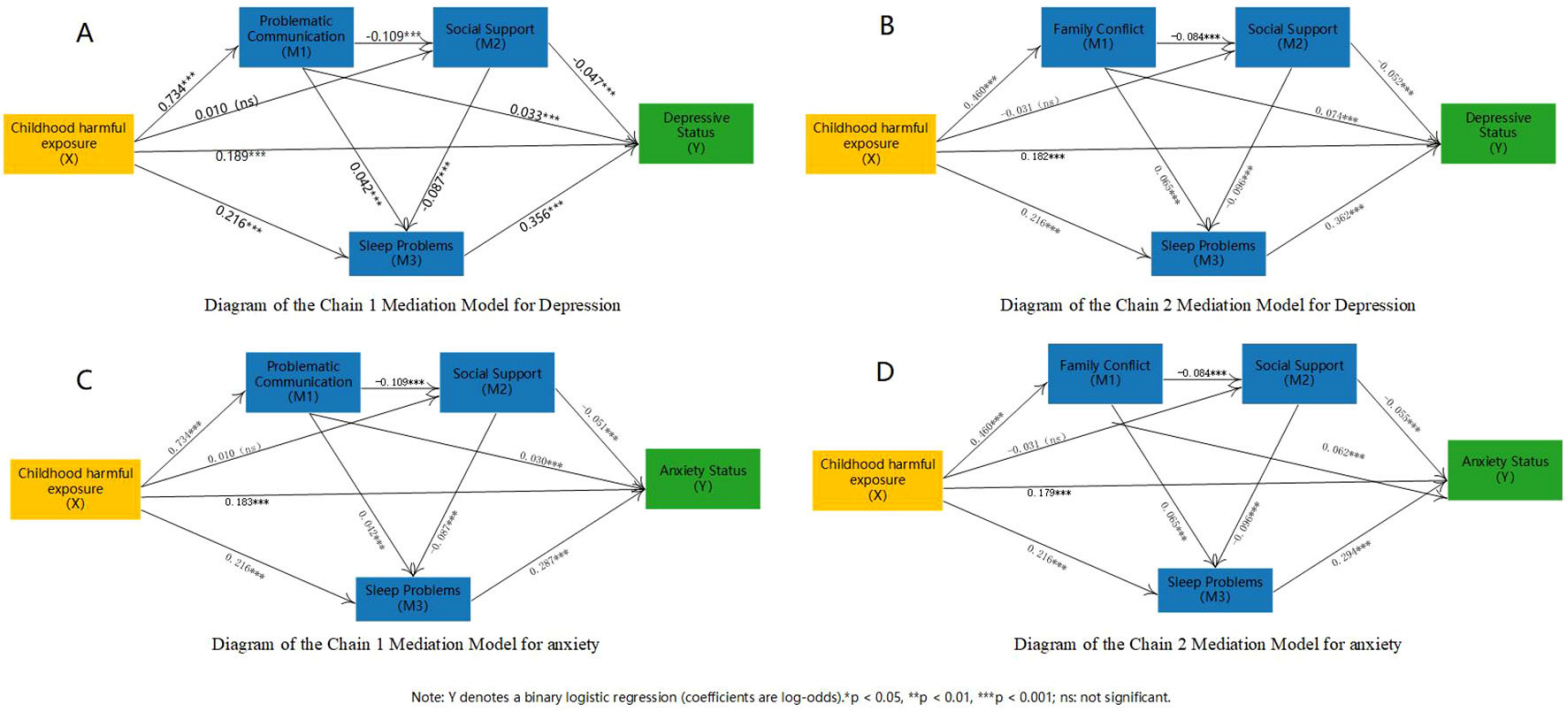
Diagram of serial mediation models for depression/anxiety status. **(A)** Chain 1 mediation model for depression, with problematic communication as M1, social support as M2, and sleep problems as M3. **(B)** Chain 2 mediation model for depression, with family conflict as M1, social support as M2, and sleep problems as M3. **(C)** Chain 1 mediation model for anxiety, with problematic communication as M1, social support as M2, and sleep problems as M3. **(D)** Chain 2 mediation model for anxiety, with family conflict as M1, social support as M2, and sleep problems as M3. Childhood harmful exposure was specified as the antecedent variable X, and depressive status/anxiety status was treated as the outcome Y.

In the main analysis, childhood harmful exposure was specified as the antecedent variable (*X*). Problematic communication or family conflict (*M*1), social support (*M*2), and sleep status (*M*3) were included as mediators, and depressive state and anxiety state were treated as outcomes (*Y*), forming two sets of models (**Figures 6A–D**). The results showed that, regardless of whether depressive state or anxiety state was used as the outcome, both the total indirect effects and the direct effects in the two model sets reached statistical significance. This indicates that the associations between childhood harmful exposure and the two outcomes included both direct components and indirect components that could be decomposed through current family interaction, social support, and sleep status.When depressive state was used as the outcome, the total indirect effects in the two models were *B*=0.117 (95% CI: 0.101–0.136, accounting for 38.24% of the total effect) and *B*=0.129 (95% CI: 0.110–0.150, accounting for 41.48%), respectively. The corresponding direct effects were *B*=0.189 (95% CI: 0.152–0.225) and *B*=0.182 (95% CI: 0.144–0.220). When anxiety state was used as the outcome, the total indirect effects of the problematic communication model and the family conflict model were *B*=0.098 (95% CI: 0.084–0.115, accounting for 34.88% of the total effect) and *B*=0.107 (95% CI: 0.089–0.126, accounting for 37.41% of the total effect), respectively, with corresponding direct effects of *B*=0.183 (95% CI: 0.148–0.219) and *B*=0.179 (95% CI: 0.143–0.216). Overall, across the four main analysis models, indirect components accounted for approximately one-third to two-fifths of the total effect, and the proportion of total indirect effects was slightly higher in the depression models than in the anxiety models.

Examining the specific indirect effects, sleep status showed the largest indirect effect magnitude across all four main analysis models. When depressive state was used as the outcome, the specific indirect effects through sleep status were *B*=0.077 and *B*=0.078, accounting for approximately 25% of the total effect. When anxiety state was used as the outcome, the corresponding effects were *B*=0.062 and *B*=0.063, which were also the largest components among the indirect pathways. These findings indicate that, in decomposing the associations between childhood harmful exposure and the two outcomes, the indirect pathway through sleep status contributed the largest share.

In comparison, the specific indirect effects of problematic communication and family conflict were smaller than those of sleep status but remained statistically significant. The indirect effects through problematic communication were *B*=0.024 and *B*=0.022, whereas the indirect effects through family conflict were *B*=0.034 and *B*=0.028. These results suggest that the associations between childhood harmful exposure and the two outcomes can be partially accounted for not only by sleep status as a relatively strong mediating component, but also by indirect components corresponding to current negative family interactions themselves. In other words, in the main analysis models, problematic communication and family conflict did not function solely as antecedent variables to sleep status; rather, each retained its own independent indirect association component.

In contrast to the above findings, when social support was examined as a single mediator, its specific indirect effect did not reach statistical significance in any of the four main analysis models. This result does not imply that social support is unrelated to the two outcomes. Rather, it suggests that after simultaneously including problematic communication/family conflict and sleep status, the indirect association attributable to social support alone was relatively small and insufficient to constitute a stable independent specific indirect effect. In other words, within this set of models, social support appeared more as a component that co-occurred with other current state variables, rather than as a mediator with a prominent independent effect size. Consistently, the full serial indirect effects were also small, accounting for less than 1% of the total effect across models. This further indicates that, under the present model specification, the larger indirect associations were primarily concentrated in the pathways “through sleep status” and “through problematic communication/family conflict, “ rather than in longer multi-step serial pathways.

Comparing depressive state and anxiety state, the overall patterns were largely consistent across the two outcomes: the total indirect effects were significant; the specific indirect effect through sleep status was the largest; problematic communication and family conflict retained independent indirect effects; and the specific indirect effect corresponding to social support alone was relatively weak. However, in terms of effect magnitude, both the total indirect effects and the specific indirect effects were slightly larger in the depression models than in the anxiety models. This suggests that, compared with anxiety state, the association between childhood harmful exposure and depressive state could be decomposed into a greater proportion of indirect association components within the current model framework.

#### 3.5.2 Supplementary mediation analysis

As a complement to the main mediation analysis, problematic communication and family conflict were further specified as antecedent variables (*X*). Social support (*M*1) and sleep problems (*M*2) were simultaneously included as mediators, with depressive state and anxiety state treated as outcomes (*Y*), in order to decompose the associations among current state variables. The results are presented in **Table 5**. Across the four supplementary models, both the direct effects and the total indirect effects reached statistical significance, and the overall patterns were consistent with those observed in the main analysis.

**Table 5:** Results of supplementary dual-mediator analysis and order-reversal sensitivity analysis.

Analysis type	X	Mediator order	Y	(c’) (95% CI)	(Indtot) (95% CI)	(IndSS) (95% CI)	(IndSLP) (95% CI)	(C_1_) (95% CI)
Supplementary analysis	PC	SS → SLP	Y1	0.065 (0.056, 0.073)	0.068 (0.063, 0.074)	0.024 (0.019, 0.028)	0.045 (0.041, 0.048)	−0.021 (−0.027, −0.015)
	PC	SS → SLP	Y2	0.058 (0.050, 0.067)	0.059 (0.054, 0.064)	0.023 (0.019, 0.027)	0.037 (0.033, 0.040)	−0.014 (−0.019, −0.008)
	FC	SS → SLP	Y1	0.177 (0.153, 0.200)	0.203 (0.187, 0.221)	0.069 (0.058, 0.080)	0.134 (0.121, 0.148)	−0.065 (−0.083, −0.048)
	FC	SS → SLP	Y2	0.157 (0.135, 0.180)	0.176 (0.162, 0.192)	0.066 (0.056, 0.077)	0.110 (0.099, 0.122)	−0.044 (−0.060, −0.028)
Order-reversal	PC	SLP → SS	Y1	0.065 (0.056, 0.073)	0.068 (0.063, 0.074)	0.024 (0.019, 0.028)	0.045 (0.041, 0.048)	0.021 (0.015, 0.027)
	PC	SLP → SS	Y2	0.058 (0.050, 0.067)	0.059 (0.054, 0.064)	0.023 (0.019, 0.027)	0.037 (0.033, 0.040)	0.014 (0.008, 0.019)
	FC	SLP → SS	Y1	0.177 (0.153, 0.200)	0.203 (0.186, 0.221)	0.069 (0.058, 0.080)	0.134 (0.121, 0.148)	0.065 (0.048, 0.083)
	FC	SLP → SS	Y2	0.157 (0.135, 0.180)	0.176 (0.161, 0.192)	0.066 (0.056, 0.077)	0.110 (0.099, 0.122)	0.044 (0.028, 0.060)

PC, problematic communication; FC, family conflict; SS, social support; SLP, sleep problems; Y1, depressive status; Y2, anxiety status;c′ represents the direct effect; Ind_tot_ represents the total indirect effect; Ind_SS_ represents the specific indirect effect through social support; and Ind_SLP_ represents the specific indirect effect through sleep problems;In the supplementary analysis, C_1_ is defined as Ind_SS_− Ind_SLP_. In the order-reversal sensitivity analysis, C_1_ is defined as Ind_SLP_− Ind_SS_; All indirect effects were estimated using the bootstrap method, and a 95% confidence interval (CI) not including 0 was considered statistically significant.

Specifically, when problematic communication was specified as the antecedent variable, the direct effect and total indirect effect in the depressive state model were *B*=0.065 and *B*=0.068, respectively; in the anxiety state model, the corresponding values were *B*=0.058 and *B*=0.059. When family conflict was specified as the antecedent variable, the direct effect and total indirect effect in the depressive state model were *B*=0.177 and *B*=0.203, respectively, whereas in the anxiety state model the corresponding values were *B*=0.157 and *B*=0.176. These results indicate that, compared with problematic communication, family conflict showed a larger overall magnitude of association with both outcomes, which is consistent with the larger indirect effects corresponding to family conflict observed in the main analysis.

Further examination of the specific indirect effects showed that, across all four supplementary models, the indirect effects through sleep problems were larger than those through social support. When problematic communication was the antecedent variable, the specific indirect effects through sleep problems and social support were *B*=0.045 and *B*=0.024, respectively, in the depressive state model, and *B*=0.037 and *B*=0.023 in the anxiety state model. When family conflict was the antecedent variable, the corresponding effects in the depressive state model were *B*=0.134 and *B*=0.069, and in the anxiety state model were *B*=0.110 and *B*=0.066. These findings indicate that the main analysis results—namely, that sleep problems corresponded to the largest indirect effect and social support corresponded to a smaller but stable indirect effect—were replicated in the supplementary models.

Overall, the supplementary mediation analyses further supported the main findings. At the level of current state variables, both problematic communication and family conflict showed significant indirect association components with depressive and anxiety states. Among these pathways, sleep problems consistently corresponded to the largest specific indirect effects, whereas social support showed smaller but stable indirect effects. At the same time, the effect sizes in the family conflict models were consistently larger than those in the problematic communication models. Although the overall patterns were similar for depressive and anxiety states, the magnitudes of the effects were generally slightly higher in the depression models than in the anxiety models.

#### 3.5.3 Order-reversal sensitivity analysis

As an additional order-reversal sensitivity analysis, the input order of the two mediators used in the supplementary mediation analysis was reversed. Accordingly, four models were constructed in the form of “problematic communication/family conflict (*X*)→ sleep problems (*M*1)→ social support (*M*2)→ depressive state/anxiety state (*Y*).” The results are presented in **Table 5**. After reversing the order of the mediators, the estimated values of the direct effects, total indirect effects, and the specific indirect effects through sleep problems and social support showed no substantive changes, and the overall pattern of results remained highly consistent with that observed in the preceding supplementary mediation analysis.

Specifically, when problematic communication was specified as the antecedent variable, the direct effect and total indirect effect in the depressive state model remained *B*=0.065 and *B*=0.068, respectively, and the corresponding values in the anxiety state model remained *B*=0.058 and *B*=0.059. For both outcomes, the specific indirect effect through sleep problems remained larger than that through social support: in the depressive state model the values were *B*=0.045 and *B*=0.024, and in the anxiety state model *B*=0.037 and *B*=0.023. Correspondingly, the mediation contrast terms changed from negative values in the original model to positive values due to the reversed definition, with values of *B*=0.021 and *B*=0.014 for the depressive and anxiety models, respectively. This again indicates that the magnitude of the indirect effect through sleep problems remained greater than that through social support.

When family conflict was specified as the antecedent variable, the results also remained stable. The direct and total indirect effects in the depressive state model were *B*=0.177 and *B*=0.203, respectively, while the corresponding values in the anxiety state model were *B*=0.157 and *B*=0.176. The specific indirect effects through sleep problems and social support were *B*=0.134 and *B*=0.069 in the depressive state model, and *B*=0.110 and *B*=0.066 in the anxiety state model. The corresponding mediation contrast terms were *B*=0.065 and *B*=0.044. These results indicate that, in the family conflict–related models, the indirect effect corresponding to sleep problems remained clearly larger than that corresponding to social support.

Overall, after reversing the mediator order, only the direction of the mediation contrast terms changed due to differences in their definitions, whereas the estimates of the main effects remained stable. In other words, at the level of current state variables, regardless of whether the antecedent variable was problematic communication or family conflict, the specific indirect effect through sleep problems consistently exceeded that through social support. Moreover, the effect sizes in the family conflict models were generally larger than those in the problematic communication models, and the effect sizes in the depression models were also slightly larger than those in the anxiety models. These findings further corroborate the results of the main mediation analysis.

## 4 Discussion

Grounded in the SPM framework, this study aimed to examine the associations of distal adversity exposure, family relational context, resource pathways, and proximal functional status with depressive and anxiety states in university students, and to further compare their shared risk structure and key points of divergence.

Based on a large sample of university students, the present study combined hierarchical logistic regression, machine learning, and mediation analyses and found that anxiety and depressive states were not associated with a single factor, but rather showed stable associations with multiple domains, including childhood harmful exposure, current family interaction, social support, proximal negative life events, and sleep status. Overall, the risk profiles of the two outcomes largely overlapped, indicating that anxiety and depression among university students share a substantial common psychosocial foundation. At the same time, the relative contributions of specific factors differed to some extent between the two outcomes, suggesting that, despite their shared vulnerability background, certain distinctions remain. Compared with basic demographic variables, indicators reflecting individuals’ developmental experiences, current relational environment, and proximal functional status showed stronger statistical associations. Existing studies have likewise suggested that the risk factors for anxiety and depression in university students arise from multilevel psychosocial sources (29, 56). Taken together, these findings indicate that internalizing symptoms in university students are better understood within a framework of jointly operating multilevel psychosocial factors.

Childhood harmful exposure was one of the most stable core variables identified in the present study. In both hierarchical logistic regression and machine-learning models, it consistently showed a significant incremental risk effect on both anxiety and depressive states. This finding has been repeatedly reported in previous research, which further suggests that its impact may persist into adolescence and early adulthood (95). Taken together, these findings highlight childhood harmful exposure as a distal indicator of adverse experience with robust and enduring associations across developmental stages. Mediation analyses further showed that a substantial proportion of the association between childhood harmful exposure and both outcomes could be accounted for by problematic communication, family conflict, social support, and sleep status. This suggests that childhood harmful exposure may exert a long-term shaping effect on subsequent relational context, resource conditions, and proximal functional manifestations. This pattern is also broadly consistent with pathway characteristics reported in previous studies. Prior research in university samples has shown that the association between childhood adversity and depression may be partially transmitted through family functioning or insomnia (62). However, such studies have generally focused on limited mediation chains and have not examined family relational context, resource pathways, and proximal functional status within the same multilevel framework. Taken together, both previous findings and the present results indicate that childhood harmful exposure is not merely a general background factor, but an important distal correlate of anxiety and depressive states in university students. This also suggests that anxiety and depressive states during the university years cannot be fully explained by current stress or recent events alone. Rather, they may also reflect earlier and cumulatively accumulated adverse experiences. This interpretation is consistent with the life-course perspective, which emphasizes that early adversity may continue to shape later mental health trajectories (96). In particular, the hierarchical models and mediation analyses in the present study jointly indicated that part of the association between childhood harmful exposure and current internalizing symptoms could be explained by the quality of current family interaction. This finding suggests that the influence of childhood harmful exposure may extend into current relational and regulatory functioning. It is also broadly in line with the direction of chain-transmission findings reported in current university-student research (62). At the same time, the present study further grounds this association in the quality of current family interaction, a domain that is more proximal to the lived relational context, thereby providing a clearer account of how early adversity may continue to shape anxiety and depressive states during the university years. A further comparison of the two outcomes showed that childhood harmful exposure had greater overall importance in the depression model than in the anxiety model. This suggests that, relative to anxiety states, depressive states may be more closely linked to longer-term and cumulative adverse experiences. Previous studies have indeed provided more extensive and more consistent evidence for the association between childhood harmful exposure and depressive outcomes, which offers an important point of reference for this pattern (97). At the same time, by examining anxiety and depression in parallel within the same risk background, the present study further showed that the two outcomes may differ in their relative centers of association despite sharing a common background. In this sense, their similarities and differences were characterized in a more fine-grained manner than in previous studies that tended to examine them separately.

Corresponding to the distal background, current negative family interaction emerged as the most salient relational dimension in the present study. Problematic communication and family conflict both showed stable and significant incremental risk effects in the hierarchical logistic regression and SHAP analyses, a pattern consistent with previous findings that family communication and family conflict are persistently associated with psychological distress, including anxiety and depression (98). Furthermore, the significance of negative family interaction was not fully replaced by social support or sleep status. This suggests that negative family interaction is not merely an accompanying factor, but rather an independent risk dimension within the current relational environment. At the same time, problematic communication and family conflict showed greater stability than positive family-functioning indicators such as cohesion and adaptability, suggesting that, in the context of multiple variables operating simultaneously, negative family interaction may exert a more direct influence on current anxiety and depressive states than positive family functioning. This finding extends previous research, which has mainly emphasized the protective role of positive family functioning through resources such as self-esteem, peer relationships, and social support (99), by further showing that, when both positive and negative dimensions of family interaction are considered within the same risk context, negative family interaction may have a more direct and pronounced association with current anxiety and depressive states than positive family functioning. More importantly, within the same risk background, positive family-functioning indicators could not substitute for the direct relational risks represented by problematic communication and family conflict, which further suggests that the influence of negative family interaction on anxiety and depression is more closely tied to the immediate tension and sustained burden embedded in the current relational context. Notably, after social support was introduced into the model, some positive family-functioning indicators became attenuated, whereas problematic communication and family conflict remained. This suggests that part of the protective association between positive family functioning and the outcomes may be expressed through support resources that individuals can perceive and mobilize; in contrast, the risk burden associated with negative family interaction retains a more direct relational-environment component and therefore cannot be fully absorbed by social support. Viewed in light of existing studies in university students (58), positive family-functioning indicators such as cohesion and adaptability appear to constitute a resource-generating background, the effects of which are largely realized through support systems that individuals can perceive and mobilize. By contrast, problematic communication and family conflict reflect direct tension and interactional dysregulation within the current relational environment; such risk burden is not equivalent to insufficient support, and therefore remained not fully absorbed by social support in the present study. Taken together, social support and negative family interaction do not operate at the same level: the former is more closely aligned with protective resources, whereas the latter retains a more direct negative relational component. This further suggests that negative family interaction has not receded into a mere background factor during the university years, but may instead constitute an important real-world relational context through which current risks of internalizing symptoms are carried and manifested.

Social support and sleep status jointly constituted the most noteworthy proximal correlates in the present study, although their statistical roles were not identical. Social support consistently emerged as a stable protective factor in the hierarchical logistic regression and SHAP analyses, indicating that it functions as an important protective resource against internalizing symptoms among university students. This finding accords with previous research suggesting that social support may operate through both a direct protective effect and a stress-buffering effect (42, 100). However, in the mediation analyses, the specific indirect effect of social support was relatively small, suggesting that its role is not primarily reflected in a single prominent pathway, but rather in its buffering influence on the overall risk structure. In other words, the practical significance of social support lies less in independently explaining how a given risk factor is translated into anxiety or depression, and more in providing a protective environment that can be perceived and mobilized by university students facing multiple adverse conditions, thereby attenuating the overall burden generated by the accumulation of multilevel risks. By contrast, the findings for sleep status were more concentrated and consistent. Across the hierarchical logistic regression and the SHAP analysis of the LightGBM model, sleep status showed the strongest and most significant association and predictive contribution. In the mediation analyses, the specific indirect effect through sleep problems was consistently greater than that through social support, and this pattern remained stable in the order-reversal sensitivity analysis. This result is broadly consistent with previous observations that sleep problems are more proximal to internalizing symptoms such as anxiety and depression (49). At the same time, the present study further suggests that sleep is not merely a functional-state indicator accompanying emotional problems, but may also represent an important proximal carrier through which multilevel risk factors—including distal adversity, negative family interaction, and insufficient resources—continue to be transmitted to current internalizing symptoms. Accordingly, compared with distal experiences or relational backgrounds that are more difficult to modify directly in the short term, sleep problems may be easier to identify, monitor, and target, and may therefore serve as a sensitive point of entry for risk screening and early support for anxiety/depression among university students. Taken together, among the multilevel risk factors examined in this study, social support appears to function more as a protective buffer against the overall risk structure, whereas sleep problems more directly and concentratively reflect the accumulated impact of these risks at the level of current functioning, thereby emerging as one of the proximal functional indicators most closely linked to anxiety and depressive states.

When the above findings are interpreted within a unified framework, the risk variables do not appear as isolated factors, but rather exhibit a relatively clear hierarchical structure and continuity. The hierarchical regression results showed that, as family interaction, social support, proximal events, and sleep status were entered sequentially, the effect of childhood harmful exposure gradually attenuated but remained significant; problematic communication and family conflict remained relatively stable after multilevel adjustment; social support retained its protective role; and sleep status provided the largest additional explanatory contribution in the final model. Multiple mediation analyses further indicated that the association between childhood harmful exposure and the outcomes could be partially decomposed through problematic communication or family conflict, social support, and sleep status. Moreover, when problematic communication and family conflict were treated as antecedent variables, their associations with anxiety and depressive states could be further reflected through social support and sleep problems. In terms of overall direction, these findings are consistent with previous observations in university students regarding chain-like transmission involving early adversity and family relationships/functioning, in that they suggest internalizing symptoms are unlikely to arise from the direct effect of a single factor, but are more likely to represent the cumulative consequences of multilevel risk and protective factors (62). However, whereas previous studies have largely focused on a limited pathway (56, 57), the present study points not merely to whether a given single pathway is present, but rather to a multilevel network of associations in which distal background, the current relational environment, resource conditions, and proximal functional problems are nested within one another. Within this structure, childhood harmful exposure corresponds primarily to the distal background of adverse experience; problematic communication and family conflict reflect the manifestation of this background at the level of current family relationships; social support reflects the protective resources available in the current environment; and sleep problems more closely represent the concentrated manifestation of these upstream adverse influences at the level of current functioning. At the same time, existing studies have tended to place greater emphasis on the prominent mediating role of social support in transmission pathways (101, 102). In the present study, however, although social support consistently emerged as an important protective factor, its specific indirect effect was not particularly prominent. This suggests that, once childhood harmful exposure, family interaction, proximal events, and sleep status are considered simultaneously, social support may function less as the core mediator in a single pathway and more as a protective background that cuts across multilevel risk factors, continuously buffering relational stress, resource depletion, and proximal functional dysregulation. By contrast, sleep problems showed more concentrated effects in both the predictive and mediation analyses, a pattern also broadly consistent with previous research viewing sleep as more proximal to internalizing symptoms such as anxiety and depression (62). This suggests that sleep more directly carries the functional dysregulation that emerges at the current stage following the accumulation of multilevel risks, and is therefore more closely linked, statistically, to anxiety and depressive states. Overall, these findings further indicate that anxiety and depression in university students are better understood as multilevel stress-process outcomes jointly shaped by distal adversity exposure, the current relational environment, resource conditions, and proximal functional problems, rather than as a simple juxtaposition of several isolated factors.

A further comparison of anxiety and depressive states showed a high degree of overlap in their core associated factors. Sleep status, childhood harmful exposure, problematic communication, family conflict, social support, and recent major negative life events repeatedly entered the set of core variables across different analytical frameworks, indicating that the two outcomes share a substantial psychosocial basis of association. This finding is broadly consistent with the overall understanding emerging from previous research in university students and from the broader literature on internalizing problems, namely, that anxiety and depression are often not driven by two entirely separate risk systems, but are more commonly expressed as different clinical manifestations arising from a shared vulnerability background (56). At the same time, the present study also showed that, within this shared risk profile, the two outcomes differed to some extent in their centers of association: anxiety states were more strongly oriented toward current relational tension, communication failure, and real-life burden, whereas depressive states were more strongly oriented toward early cumulative adverse experiences and long-term resource depletion. More specifically, problematic communication, family conflict, and open communication played a more prominent role in the anxiety models. This pattern is broadly consistent with previous findings suggesting that the current relational environment and interactional dysregulation are more likely to be directly linked to anxiety-like symptoms (103), and further suggests that anxiety states may be more sensitive than depressive states to tension, uncertainty, and communication failure in the current relational environment. By contrast, depressive states retained a more evident association with childhood harmful exposure (104), indicating that depression is not only related to current difficulties, but may also more readily carry the vulnerability background and the consequences of resource depletion accumulated from earlier adverse experiences. Although some previous studies have separately emphasized the importance of current relational factors for anxiety or of early adversity for depression (96), most have examined these issues within a single outcome and have not directly tested the commonalities and differences between anxiety and depression in parallel. By examining them within the same multilevel psychosocial framework, the present study not only confirmed that the two outcomes share a common vulnerability background, but also further showed that, within a shared risk structure, anxiety and depression are not simply homogeneous conditions; rather, they are characterized by two different centers of risk, one oriented toward current relational tension and the other toward distal cumulative adversity. This implies that the commonality of internalizing symptoms does not preclude structural differences within them. On the contrary, it is precisely by identifying such shifts in emphasis within a shared background that one can more accurately understand why anxiety and depression are both highly co-occurring and yet not fully identical. These findings also suggest that the practical points of entry for addressing anxiety and depression should not be entirely the same. For individuals whose presentation is more anxiety-prone, the identification and regulation of current relational tension, communication failure, and real-life burden may be particularly important. For those whose presentation is more depression-prone, greater attention may need to be paid to the continuing influence of early adverse experiences and long-term resource depletion.

From a practical perspective, the implications of the present study extend beyond simply indicating that anxiety and depression in university students have a multilevel psychosocial basis. More importantly, they suggest that, in the processes of identification, assessment, and support, university mental health services should not rely on single-layered judgments based solely on surface emotional manifestations or recent stressors, but should instead incorporate multilevel information, including distal adversity exposure, the current relational environment, protective resources, and proximal functional status. In this regard, childhood harmful exposure, negative family interaction, insufficient social support, and sleep problems may all be regarded as key indicators with high screening value, although their implications are not the same: the first primarily signals a persistent vulnerability background, family interaction reflects the risk load embedded in the current relational context, social support indicates the level of mobilizable protective resources, and sleep is more proximal to current functional dysregulation. Based on these findings, the identification and support of anxiety and depressive states in university students may be better grounded in the integration of multilevel information, the recognition of dominant risk patterns, and stratified attention. Correspondingly, support strategies should place greater emphasis on the timely improvement of proximal functional status, the buffering of current relational stress, the reinforcement of protective resources, and sustained attention to distal vulnerability backgrounds, rather than remaining at a uniform and single-layered level of response.

## 5 Limitations and future directions

Several limitations of the present study should be acknowledged. First, this study employed a cross-sectional design. Although the hierarchical models and mediation analyses provided statistical support for the proposed chain-like direction among variables, the inherent limitations of cross-sectional data (105) preclude rigorous confirmation of causal direction and temporal ordering, and are particularly unfavorable for examining the dynamic transmission of the stress process across different stages. Current research has likewise indicated that a better understanding of changes in university students’ mental health and their underlying mechanisms still requires more longitudinal evidence (106). Future studies should therefore consider longitudinal follow-up or cross-lagged designs to more rigorously examine the temporal relationships among childhood adversity, family relationships, social support, sleep, and anxiety/depression.Second, the main variables in this study were derived from self-report questionnaires. Methodological research has shown that self-reported data may be influenced by response bias, social desirability bias, and differences in frames of reference, thereby introducing common method bias and reporting bias (107, 108). Future research may therefore benefit from incorporating objective sleep indicators, more refined assessments of stress exposure, and data from multiple informants in order to enhance the robustness of the findings. Third, the sampling frame of the present study was subject to certain limitations. Previous studies have noted that research on university students’ mental health commonly faces problems such as limited sample representativeness, participation bias, and overrepresentation of specific groups (109, 110). In addition, student samples are often more homogeneous than the general population, and differences across regions, institutional types, and cultural contexts may further constrain the generalizability of the findings (109). Accordingly, the present results still require replication across different regions, educational settings, and sociocultural environments. In addition, although the present study found a relatively marked shared risk structure for anxiety and depression, together with certain differences in the strength of specific associations, the similarities and differences between the two outcomes at deeper cognitive and developmental-mechanism levels have not yet been adequately examined (111, 112). Future research may therefore compare the common mechanisms and specific transmission pathways of anxiety and depression within the same variable system in order to achieve a more fine-grained theoretical understanding.Despite these limitations, the present study, grounded in the SPM framework, integrated multilevel factors spanning distal adversity exposure, family relational context, resource pathways, and proximal functional status, and identified key nodes through converging evidence from hierarchical logistic regression, machine learning, and mediation analyses. The findings thus provide a relatively clear empirical basis for understanding the multilevel psychosocial mechanisms underlying anxiety/depression in university students and for informing the prioritization of more operationally relevant intervention targets.

## 6 Conclusion

Based on a large sample of university students, and integrating the findings from hierarchical logistic regression, machine learning, and mediation analyses, this study demonstrates that anxiety and depressive states cannot be explained by a single factor. Instead, they are stably associated with multiple factors, including childhood harmful exposure, problematic communication and family conflict, insufficient social support, recent major negative life events, and sleep problems. Among these factors, childhood harmful exposure primarily reflects a long-term vulnerability background formed early in life; problematic communication and family conflict correspond directly to persistent stress within the current family environment; insufficient social support reflects a lack of accessible protective resources; and sleep problems represent the most direct and prominent proximal functional indicator associated with current anxiety and depressive states.The results further suggest that these factors exhibit a sequential statistical association structure in which distal adverse exposure, current family relational stress, resource insufficiency, and proximal sleep impairment progressively approach emotional outcomes. At the same time, although anxiety and depressive states share a highly similar risk profile, certain differences remain: anxiety is more prominently associated with current relational tension, communication breakdown, and real-life pressures, whereas depression shows a stronger association with long-term cumulative adverse experiences such as childhood harmful exposure.These findings indicate that mental health efforts in universities should not remain limited to symptom screening or the identification of recent stressors. Instead, childhood harmful exposure, negative family interactions, social support, and sleep problems should be incorporated as key elements in assessment frameworks. Establishing a more hierarchical approach to risk identification and intervention may facilitate the earlier detection of students who are truly at sustained high risk and improve the precision of identifying and supporting anxiety and depressive problems.

## Data Availability

The raw data supporting the conclusions of this article will be made available by the authors, without undue reservation.
